# Activation of pruritogenic TGR5, MrgprA3, and MrgprC11 on colon-innervating afferents induces visceral hypersensitivity

**DOI:** 10.1172/jci.insight.131712

**Published:** 2019-10-17

**Authors:** Joel Castro, Andrea M. Harrington, TinaMarie Lieu, Sonia Garcia-Caraballo, Jessica Maddern, Gudrun Schober, Tracey O’Donnell, Luke Grundy, Amanda L. Lumsden, Paul Miller, Andre Ghetti, Martin S. Steinhoff, Daniel P. Poole, Xinzhong Dong, Lin Chang, Nigel W. Bunnett, Stuart M. Brierley

**Affiliations:** 1 Visceral Pain Research Group, College of Medicine and Public Health, Flinders University, Bedford Park, South Australia, Australia.; 2 Hopwood Centre for Neurobiology, Lifelong Health Theme, South Australian Health and Medical Research Institute (SAHMRI), North Terrace, Adelaide, South Australia, Australia.; 3 Centre for Nutrition and Gastrointestinal Diseases, Discipline of Medicine, University of Adelaide, Adelaide, South Australia, Australia.; 4 Monash Institute of Pharmaceutical Sciences, Monash University, Parkville, Victoria, Australia.; 5 AnaBios Corporation, San Diego, California, USA.; 6 Department of Dermatology and Dermatology Immunology Institute, Hamad Medical Corporation, Doha, Qatar.; 7 Department of Dermatology, Weill Cornell Medicine-Qatar and Weill Cornell University, New York, New York, USA.; 8 School of Medicine Qatar University, Doha, Qatar.; 9 Australian Research Council Centre of Excellence in Convergent Bio-Nano Science and Technology, Monash University, Parkville, Victoria, Australia.; 10 Department of Anatomy and Neuroscience, University of Melbourne, Parkville, Victoria, Australia.; 11 The Solomon H. Snyder Department of Neuroscience, Center for Sensory Biology, School of Medicine, Howard Hughes Medical Institute, Johns Hopkins University, Baltimore, Maryland, USA.; 12 G. Oppenheimer Centre for Neurobiology of Stress and Resilience, David Geffen School of Medicine at UCLA, UCLA, Los Angeles, California, USA.; 13 Department of Pharmacology and Therapeutics, University of Melbourne, Parkville, Victoria, Australia.; 14 Department of Surgery and; 15 Department of Pharmacology, Columbia University, New York, New York, USA.

**Keywords:** Gastroenterology, Neuroscience, G-protein coupled receptors, Ion channels, Pain

## Abstract

Itch induces scratching that removes irritants from the skin, whereas pain initiates withdrawal or avoidance of tissue damage. While pain arises from both the skin and viscera, we investigated whether pruritogenic irritant mechanisms also function within visceral pathways. We show that subsets of colon-innervating sensory neurons in mice express, either individually or in combination, the pruritogenic receptors *Tgr5* and the Mas-gene–related GPCRs *Mrgpra3* and *Mrgprc11*. Agonists of these receptors activated subsets of colonic sensory neurons and evoked colonic afferent mechanical hypersensitivity via a TRPA1-dependent mechanism. In vivo intracolonic administration of individual TGR5, MrgprA3, or MrgprC11 agonists induced pronounced visceral hypersensitivity to colorectal distension. Coadministration of these agonists as an “itch cocktail” augmented hypersensitivity to colorectal distension and changed mouse behavior. These irritant mechanisms were maintained and enhanced in a model of chronic visceral hypersensitivity relevant to irritable bowel syndrome. Neurons from human dorsal root ganglia also expressed *TGR5*, as well as the human ortholog *MrgprX1*, and showed increased responsiveness to pruritogenic agonists in pathological states. These data support the existence of an irritant-sensing system in the colon that is a visceral representation of the itch pathways found in skin, thereby contributing to sensory disturbances accompanying common intestinal disorders.

## Abstract 2

TGR5, MrgprA3, and MrgprC11, receptors normally associated with pruritis, are expressed by colonic afferents and, when activated, induce visceral hypersensitivity relevant to irritable bowel syndrome.

## Graphical abstract

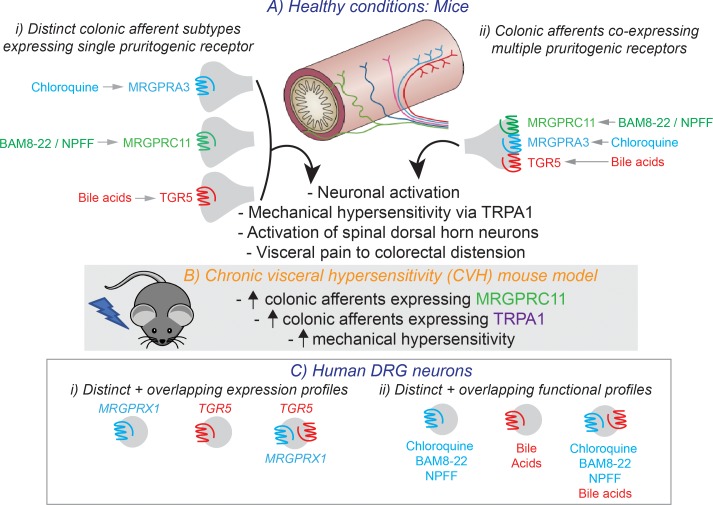

## Introduction

Itch, like pain, is a protective mechanism necessary for survival (1). Itch induces protective scratching that removes harmful irritants from the skin, while pain initiates withdrawal from and avoidance of noxious stimulants. Itch and pain are detected by primary sensory dorsal root ganglion (DRG) neurons that project from peripheral tissues into the dorsal horn (DH) of the spinal cord, where they release transmitters that excite spinal neurons (2). In the skin, histamine-dependent mechanisms contribute to itch; however, several distinct histamine-independent itch mechanisms have also been described. One involves the Mas-gene–related GPCR family, which includes MrgprA3 and MrgprC11 (2–5). Another mechanism involves the bile acid receptor TGR5, also known as GPR130 or GpBAR1 (6).

MrgprA3 and MrgprC11 are expressed by subsets of sensory DRG neurons innervating the skin (7, 8). Activation of MrgprA3 by the antimalarial drug chloroquine (CQ) (8), or MrgprC11 activation by the endogenous pruritogen, bovine adrenal medulla 8-22 peptide (BAM8-22), induces itch (3, 9). Mice lacking a cluster of *Mrgpr* genes (*Mrgpr-cluster^–/–^*) display significant deficits in itch induced by either CQ or BAM8-22, but — crucially — not itch induced by histamine (8). TGR5 is also expressed by a subpopulation of peptidergic DRG neurons and activation of TGR5 by bile acids, such as deoxycholic acid (DCA) or oleanolic acid (OA), induces neuronal excitability and also induces itch in mice (6, 10). These effects are lost in *Tgr5^–/–^* mice and are exacerbated in mice overexpressing TGR5 (*Tgr5-Tg*), potentially explaining why pruritus is observed in patients with cholestatic liver disease, where circulating bile acids are increased by 20-fold (6). However, it remains unclear if both TGR5 and MRGPR mechanisms coexist within the same DRG neuronal populations or whether they exist in, and therefore recruit, distinct populations of DRG neurons.

In the colon, afferent sensitization occurs via a variety of processes (11), including histamine-dependent mechanisms (12); however, other pathways are also likely involved. For example, increased fecal levels of bile acids have been implicated as the cause of diarrhea in a subset of patients with irritable bowel syndrome (IBS) (13), while abdominal pain and cramping are known side-effects of CQ treatment (14). Therefore, as pain arises from both the skin and viscera, we wondered whether pruritogenic irritant mechanisms identified within the skin have analogous pathways within the viscera. This is important, as chronic abdominal pain or discomfort associated with altered bowel habits are key symptoms of IBS, a prevalent functional gastrointestinal disorder affecting ~11% of the global population (15). These symptoms significantly affect patient quality of life and are notoriously difficult to treat. Although the pathophysiology of IBS is not completely understood, hallmarks of IBS include hypersensitivity to mechanical events within the intestine in the absence of overt pathology to the intestinal mucosa, resulting in allodynia and hyperalgesia (15). While sensitization and neuroplasticity of colonic afferent pathways has been implicated in the development and maintenance of chronic abdominal pain in IBS (15–17), the underlying mechanisms contributing to afferent sensitization remain incompletely understood (18). We hypothesized that MrgprA3-, MrgprC11- and TGR5-dependent mechanisms could be important mechanisms in this process.

The aim of this study was to determine if colonic afferents express TGR5, MrgprC11, and MrgprA3, and if so, whether they are present in distinct or overlapping subsets of colon-innervating DRG neurons. We also aimed to determine if agonists for TGR5, MrgprC11, and MrgprA3 induce changes in colonic sensory signaling in vitro and ex vivo and whether this translated to altered visceral sensitivity and behavior in vivo. We determined if such mechanisms were present, or indeed augmented, in a model of chronic visceral hypersensitivity (CVH) relevant to IBS. Crucially, we aimed to translate these findings to humans by using colonic biopsies and DRG sensory neurons from human donors to confirm expression profiles and functional mechanisms.

We demonstrate that *Tgr5*, *MrgprA3*, and *MrgprC11* are all expressed by colon-innervating DRG neurons, in both distinct and overlapping subsets of sensory DRG neurons, and their activation causes fundamental signaling changes within colonic afferent pathways in healthy and disease states. In human DRG neurons, TGR5 and MrgprX1 also display both distinct and overlapping molecular and functional expression profiles, with increased responsiveness to pruritogens in sensitized states.

## Results

### Agonists for TGR5, MrgprA3, and MrgprC11 evoke mechanical hypersensitivity in colonic afferents.

**Figure 1:**
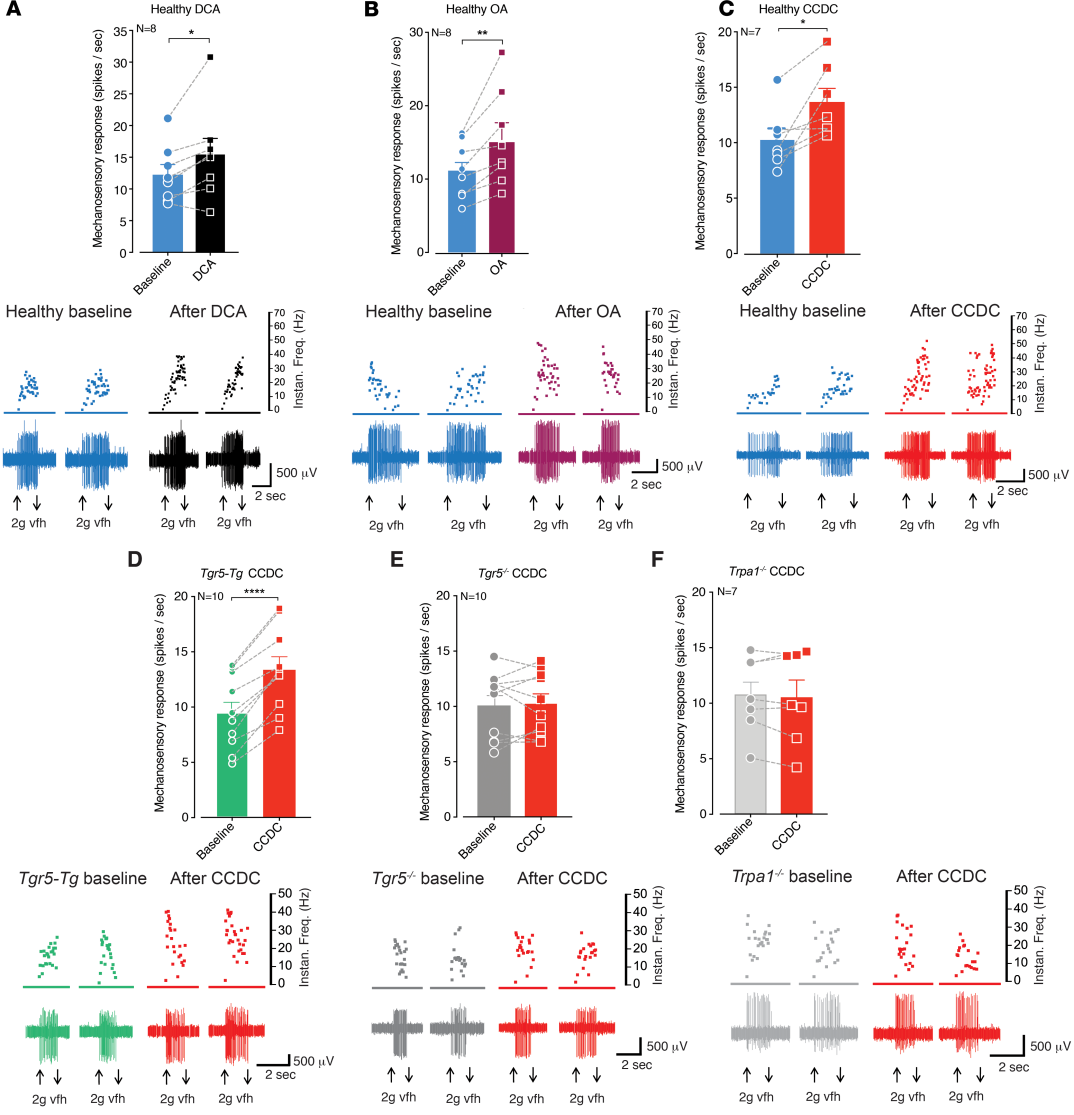
Agonists for TGR5 evoke mechanical hypersensitivity in colonic afferents. (**A**) Application of the TGR5 agonist deoxycholic acid (DCA; 100 μM) to the colonic mucosa for 5 minutes resulted in subsequent mechanical hypersensitivity of colonic nociceptors from healthy mice (*P < 0.05, *N* = 8). Dots represent values from individual afferents before and after DCA application. Lower panel shows representative recordings from a single colonic afferent nerve fiber responding to a 2 g von Frey hair (vfh) before and after incubation with DCA. (**B**) Application of the TGR5 agonist oleanolic acid (OA; 100 μM for 5 minutes) also caused mechanical hypersensitivity in nociceptors from healthy mice (**P < 0.01, *N* = 8). (**C**) The potent synthetic TGR5 agonist CCDC (100 μM for 5 minutes) also evoked mechanical hypersensitivity of colonic nociceptors from healthy mice (*P < 0.05, *N* = 7). (**D**) CCDC-induced (100 μM) mechanical hypersensitivity was enhanced in colonic nociceptors from mice overexpressing TGR5 (*Tgr5-Tg*, ****P < 0.0001, *N* = 10), but (**E**) was not observed in colonic nociceptors from *Tgr5*^–/–^ mice (*P* > 0.05, *N* = 10). (**F**) Furthermore, CCDC-induced (100 μM) mechanical hypersensitivity was not observed in colonic nociceptors from *Trpa1*^–/–^ mice (*P* > 0.05, *N* = 7), suggesting a key interaction between TGR5 and TRPA1 in the mechanical hypersensitivity evoked by TGR5 activation. Data represent mean ± SEM. *P* values determined by paired *t* tests.

**Figure 2:**
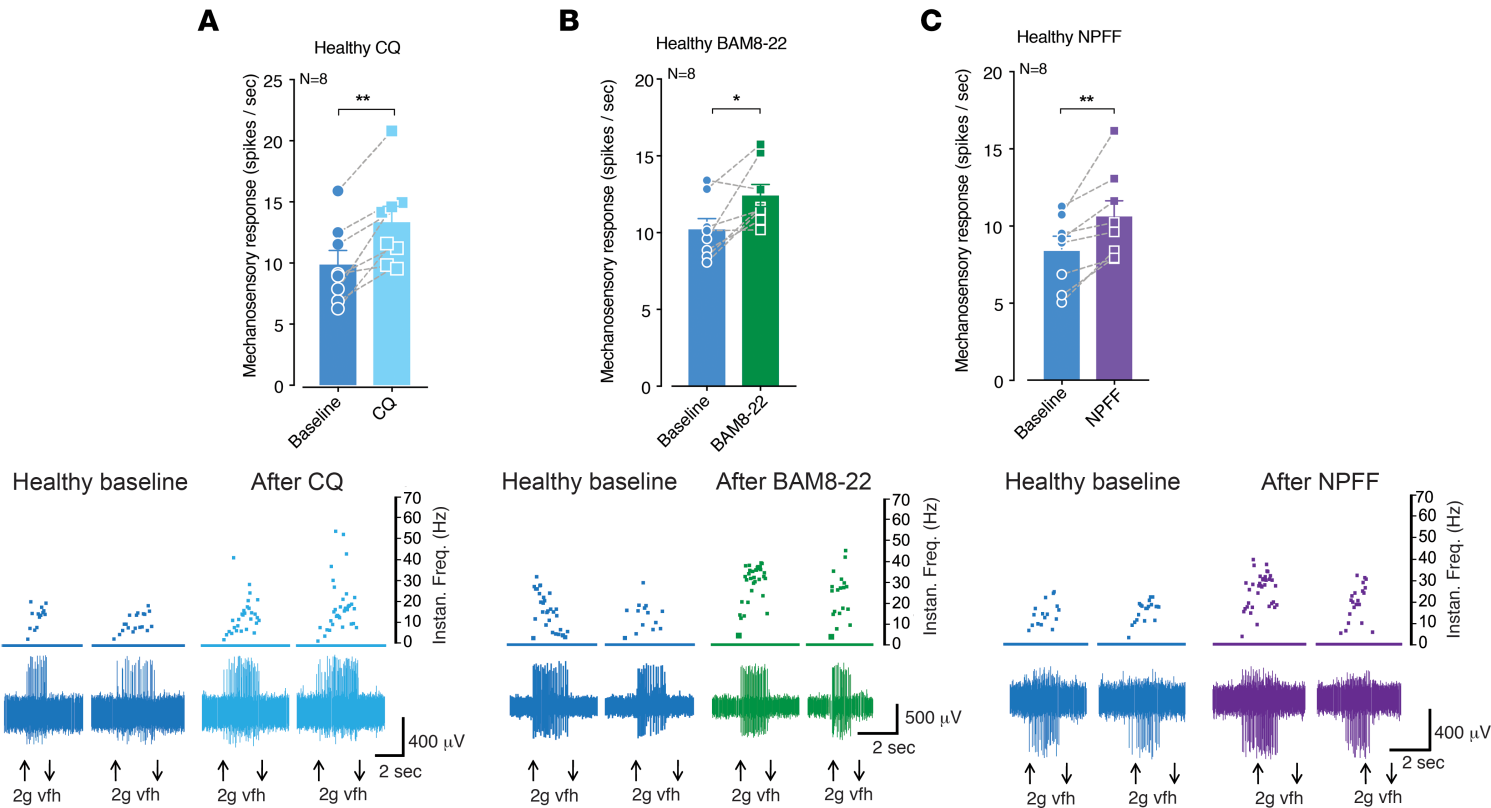
Agonists for MRGPRA3 and MRGPRC11 evoke mechanical hypersensitivity in colonic afferents. (**A**) Application of the MrgprA3 agonist chloroquine (CQ; 10 μM for 5 minutes) resulted in subsequent mechanical hypersensitivity of colonic nociceptors from healthy mice (**P < 0.01, *N* = 8). (**B**) The MrgprC11 agonist BAM8-22 (20 μM for 5 minutes) also caused mechanical hypersensitivity in nociceptors from healthy mice (*P < 0.05, *N* = 8). (**C**) Application of the combined MrgprC11/MrgprA4 agonist neuropeptide FF (NPFF; 5 μM for 5 minutes) also evoked mechanical hypersensitivity of colonic nociceptors from healthy mice (**P < 0.01, *N* = 8). Data represent mean ± SEM. *P* values determined by paired *t* tests.

In order to determine if pruritogenic receptors have a functional role in colonic sensory function, we made ex vivo recordings of colonic afferents from mice. Application of the TGR5 agonists DCA, OA, and 3-(2-chlorophenyl)-N-(4-chlorophenyl)-N,5-dimethyl-4-isoxazolecarboxamide (CCDC) evoked mechanical hypersensitivity in colonic afferent endings from healthy mice (Figure 1, A–C). Closer examination of individual afferent responses showed that some afferents were unaffected by TGR5 activation, whereas others displayed pronounced mechanical hypersensitivity (Figure 1, A–C), suggesting that TGR5 is expressed by specific subpopulations of colonic afferents. Notably, the effects of CCDC were exacerbated in colonic afferents from mice overexpressing TGR5 (*Tgr5-Tg*, Figure 1D) and lost in afferents from *Tgr5*-null mutant (*Tgr5*^–/–^) mice (Figure 1E). As TGR5 activates transient receptor potential ankyrin 1 (TRPA1) to induce itch (10), TRPA1 mediates nociceptive responses (19, 20), and we have previously shown that TRPA1 is a key integrator for the induction of mechanical hypersensitivity in colonic afferents by a variety of mediators (21–23), we applied CCDC to colonic afferents from *Trpa1^–/–^* mice. Correspondingly, we found that CCDC failed to induce mechanical hypersensitivity in afferents from *Trpa1^–/–^* mice (Figure 1F), suggesting that a key integration between TGR5 and TRPA1 exists in colonic afferents. In terms of Mrgpr signaling, CQ — an agonist of MrgprA3 — also evoked mechanical hypersensitivity in colonic afferents from healthy mice (Figure 2A). Similarly, the MrgprC11 agonist BAM8-22 (Figure 2B) and the combined MrgprC11/MrgprA4 agonist neuropeptide FF (NPFF) (Figure 2C) also evoked colonic afferent mechanical hypersensitivity. As observed with DCA, OA, and CCDC, closer examination of individual afferent responses showed that some afferents were unaffected by CQ, BAM8-22, or NPFF, whereas others displayed pronounced mechanical hypersensitivity (Figure 2, A–C), suggesting MrgprA3 and MrgprC11 expression on specific subpopulations of colon-innervating afferents.

**Figure 3:**
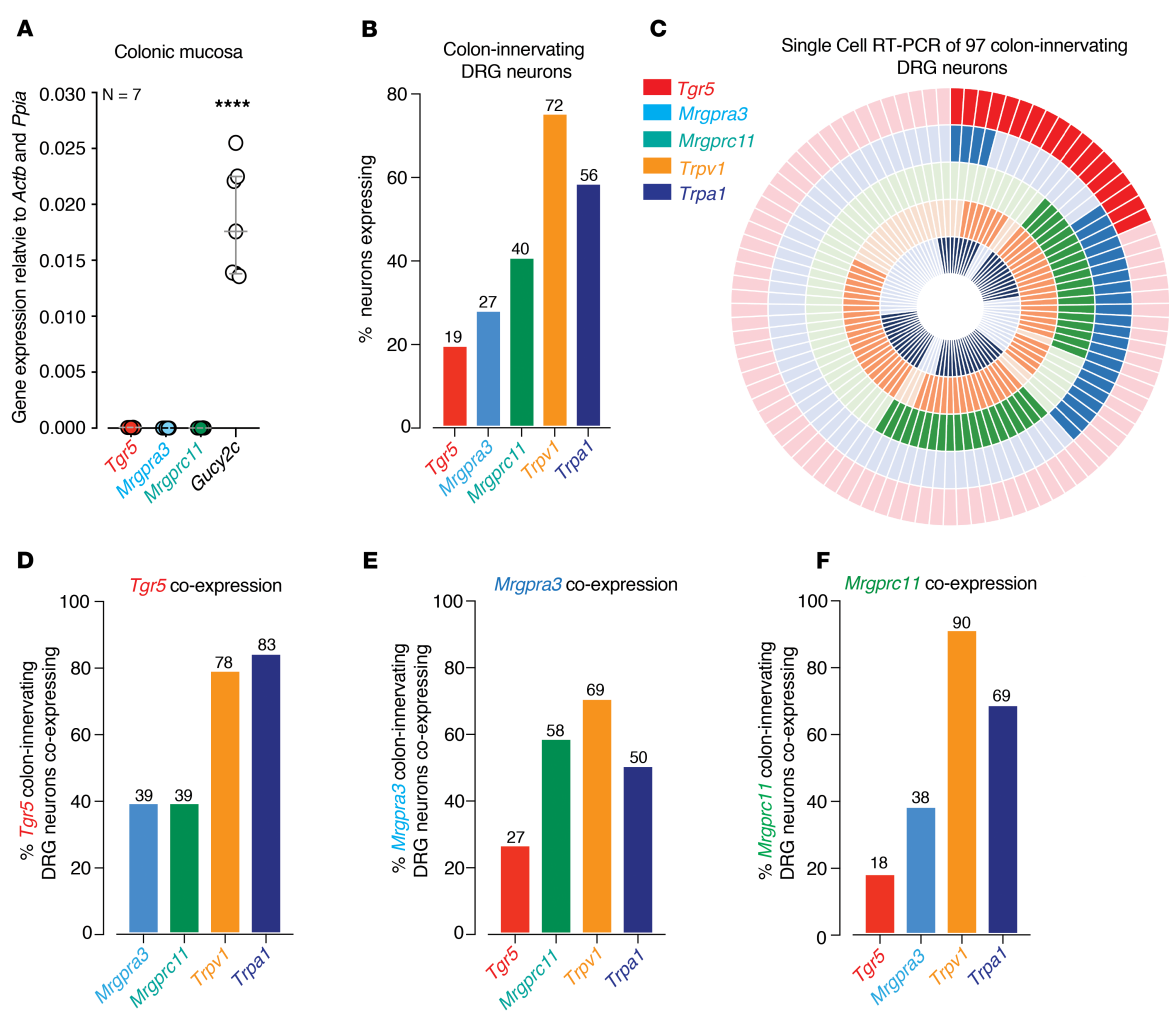
*Tgr5*, *Mrgprc11*, and *Mrgpra3* are expressed in both distinct and overlapping subpopulations of colon-innervating DRG neurons. (**A**) qPCR analysis showing low mRNA abundance for *Tgr5*, *Mrgpra3*, and *Mrgprc11* in the colonic mucosa compared with a known epithelial target *gucy2c* (guanylate cyclase-C, ****P < 0.0001, *N* = 7; each dot represents data from an individual mouse). (**B**) Single-cell RT-PCR of 97 retrogradely traced colon-innervating DRG neurons (from *N* = 5 mice) reveals that subpopulations express transcripts encoding *Tgr5* (19%), *Mrgpra3* (27%), *Mrgprc11* (40%), *Trpv1* (72%), and *Trpa1* (56%). (**C**) Donut plot showing expression and coexpression of genes encoding *Tgr5*, *Mrgpra3*, *Mrgprc11*, *Trpv1*, and *Trpa1* in 97 individual retrogradely traced colon-innervating DRG neurons. Each color represents an individual gene with expression marked by bold shading. *Tgr5* is represented in the outer ring, with *Trpa1* in the inner ring. Individual neurons are arranged radially, such that coexpression of genes in a single neuron can be easily identified running from outside to inside. Some neurons express all targets, while other neurons express combinations of targets. (**D–F**) Group data showing that (**D**) *Tgr5*, (**E**) *Mrgpra3*, and (**F**) *Mrgprc11* are expressed individually within subpopulations of colon-innervating DRG neurons and also coexpress together in other subpopulations. For example, of the *Tgr5*-expressing colon-innervating DRG neurons from healthy mice, 39% coexpress *MrgprA3* and 39% coexpress *MrgprC11*. Furthermore, *Tgr5*, *Mrgpra3*, and *Mrgprc11* also coexpress with *Trpv1* (69%–90%) and *Trpa1* (50%–83%). Data in **A** represent mean ± SEM, with *P* values determined by 1-way ANOVA with Tukey’s multiple comparison tests.

To determine the mechanisms by which TGR5 and Mrgpr agonists induce colonic afferent hypersensitivity, we confirmed expression of *Tgr5*, *Mrgpra3*, and *Mrgprc11* mRNA using quantitative PCR (qPCR) and single-cell reverse transcription PCR (RT-PCR) studies of colonic DRG neurons. Analysis of colonic mucosa from healthy mice by qPCR revealed that *Tgr5*, *Mrgpra3*, and *Mrgprc11* mRNA were all expressed in low abundance, particularly when compared with a known epithelial target such a guanylate cyclase-C (*Gucy2c*; Figure 3A). To determine if *Tgr5*, *MrgprA3*, and *MrgprC11* were expressed by colonic afferent DRG neurons, we performed single-cell RT-PCR from retrogradely traced colon-innervating DRG neurons. We also compared expression profiles with *Trpv1* and *Trpa1*, key channels involved in colonic afferent function (11, 22, 24). Of 97 individual neurons, 19% expressed *Tgr5*, 27% expressed MrgprA3, and 40% expressed MrgprC11 (Figure 3B). In comparison, *Trpv1* and *Trpa1* were expressed by 72% and 56% of colon-innervating DRG neurons, respectively (Figure 3B). These findings indicate that these pruritogenic receptors are expressed on sensory neurons innervating the colon, correlating well with our observation that subpopulations of afferents display mechanical hypersensitivity following application of the respective TGR5 and MRGPR agonists. Interestingly, we found that *Tgr5*, *Mrgpra3*, and *MrgprC11* were expressed either within the same colon-innervating DRG neuron or within separate subtypes of neurons (Figure 3C). For example, of the *Tgr5*-expressing population of colonic DRG neurons, 39% also expressed *Mrgpra3* and 39% *Mrgprc11* (Figure 3D). Of the *Mrgpra3* expressing population, 27% coexpressed *Tgr5*, while 58% coexpressed *Mrgprc11* (Figure 3E). Moreover, of the *Mrgprc11*-expressing neurons, 18% coexpressed *Tgr5*, with 38% coexpressing *Mrgpra3* (Figure 3F). Overall, *Tgr5*, *Mrgpra3*, and *Mrgprc11* were heavily coexpressed with both *Trpv1* (69%–90%) and *Trpa1* (50%–83%; Figure 3, C–F). Therefore, *Tgr5*, *Mrgpra3*, and *Mrgprc11* are expressed by both distinct and overlapping subsets of colon-innervating DRG neurons, the majority of which coexpress *Trpa1*, *Trpv1*, or both channels (Figure 3, C–F).

### Agonists for TGR5, MRGPRA3, and MRGPRC11 activate multiple populations of isolated colon-innervating sensory neurons.

**Figure 4:**
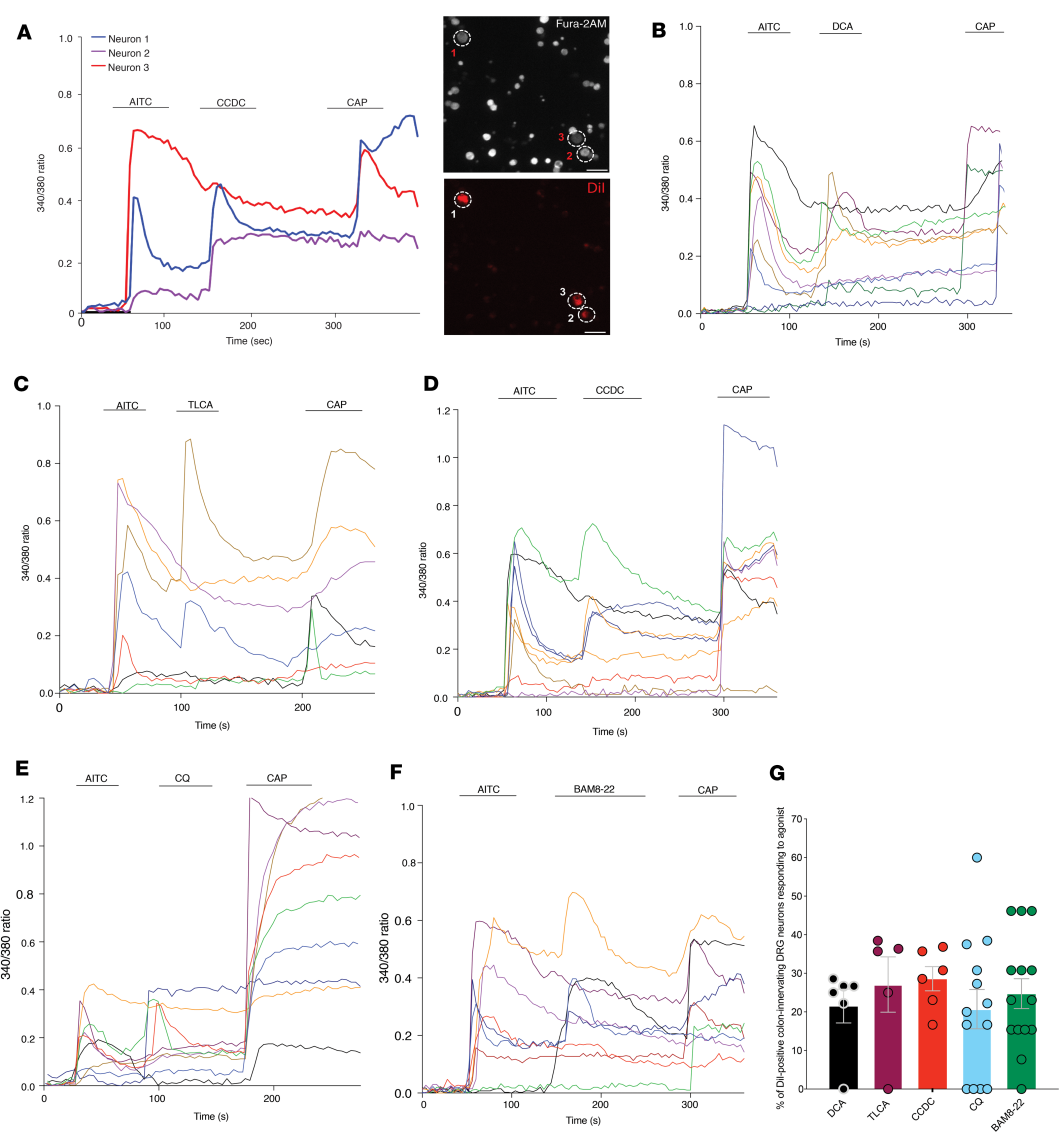
Colon-innervating DRG neurons respond to pruritogenic agonists for TGR5, MrgprA3, and MrgprC11. (**A**) Representative Ca^2+^ responses to the application of the TRPA1 agonist allyl isothiocyanate (AITC; 100 μM), the TGR5 agonist CCDC (100 μM), and the TRPV1 agonist capsaicin (CAP; 1 μM) in 3 DiI-labeled DRG neurons retrogradely labeled from the mouse colon. Right panels show Fura-2 AM image of all cells within the field of view and the 3 DiI-labeled colon-innervating DRG neurons recorded from the left panel. Scale bar: 20μm. (**B–F**) Representative traces of Ca^2+^ responses in DiI-labeled colon-innervating DRG neurons to sequential application of AITC (100 μM), the TGR5 agonists (**B**) deoxycholic acid (DCA; 100 μM), (**C**) taurolithocholic acid (TLCA; 100 μM), and (**D**) CCDC (100 μM), or the (**E**) MrgprA3 agonist chloroquine (CQ; 10 μM) and the (**F**) MrgprC11 agonist BAM8-22 (2 μM), followed by capsaicin (1 μM) and KCl (50 mM; not shown). DCA, TLCA, CCDC, CQ, and BAM8-22 all activated subpopulations of colon-innervating DRG neurons with varying functional coexpression with TRPA1 (AITC) and/or TRPV1 (capsaicin). (**G**) Group data showing the percentage of colon-innervating DRG neurons responding to DCA (61 neurons tested), TLCA (93 neurons tested), CCDC (93 neurons tested), CQ (94 neurons tested), and BAM8-22 (110 neurons tested). Each dot represents data from individual coverslips from a total of 6 mice. Data presented are mean ± SEM.

In order to confirm the results of our single-cell RT-PCR at a functional level, and to investigate the roles of pruritogenic irritants in activating colon-innervating DRG neurons, we measured intracellular calcium ([Ca^2+^]_i_) using Fura-2 AM in response to application of TGR5 and Mrgpr agonists (Figure 4, A–G). In previous studies, we have shown that DCA-evoked Ca^2+^ transients in DRG neurons are generated by a TGR5-dependent process (6, 10). Here, we show in colon-innervating DRG neurons from healthy mice, the TGR5 agonists DCA (Figure 4B), taurolithocholic acid (TLCA; Figure 4C), and CCDC (Figure 4D) all caused a robust increase in [Ca^2+^]_i_ in subpopulations of colon-innervating DRG neurons. Overall, 21.5% ± 4.4% of colonic DRG neurons responded to DCA, 27.1% ± 7.2% responded to TLCA, and 28.6% ± 3.1% responded to CCDC (Figure 4, B–D, and G). Furthermore, the MrgprA3 agonist CQ activated 20.7% ± 5.1% of colon-innervating DRG neurons (Figure 4, E and G), with the MrgprC11 agonist BAM8-22 activating 24.7% ± 3.8% of neurons (Figure 4, F and G).

To further characterize these subpopulations, we quantified the proportion of colon-innervating DRG neurons that responded to TGR5 (CCDC, DCA, TLCA), MrgprA3 (CQ), or MrgprC11 (BAM8-22), along with TRPA1 (allyl isothiocyanate; AITC) and TRPV1 (capsaicin) agonists (Figure 4, A–F). Overall, 6%–11% of colonic DRG neurons responded to the TGR5 agonists (either DCA, TLCA, or CCDC), AITC, and capsaicin, suggesting functional coexpression of TGR5, TRPA1, and TRPV1 (Supplemental Figure 1; supplemental material available online with this article; https://doi.org/10.1172/jci.insight.131712DS1). Furthermore, 7%–8% of colon-innervating DRG neurons responded to the TGR5 agonists and AITC, but not capsaicin (suggesting coexpression of TGR5 and TRPA1), with only about 2%–6% of neurons responding to the TGR5 agonists and capsaicin alone (coexpression of TGR5 and TRPV1; Supplemental Figure 1). Similarly, ~9% of colon-innervating DRG neurons responded to CQ, AITC, and capsaicin (MrgprA3, TRPA1, TRPV1 coexpression), with 4% responding to CQ and AITC but not capsaicin, and 7% responding to CQ and capsaicin but not AITC (Supplemental Figure 1). Finally, ~10% of colon-innervating DRG neurons responded to BAM8-22, AITC, and capsaicin (MrgprC11, TRPA1, TRPV1 coexpression), with 8% responding to BAM8-22 and AITC but not capsaicin, and 6% responding to BAM8-22 and capsaicin but not AITC (Supplemental Figure 1). These results support a functional role for TGR5, MrgprA3, and MrgprC11 in overlapping and distinct populations of TRPA1- and/or TRPV1-expressing colon-innervating DRG neurons.

### In vivo intracolonic administration of pruritogenic agonists increases signaling within the DH of the spinal cord.

**Figure 5:**
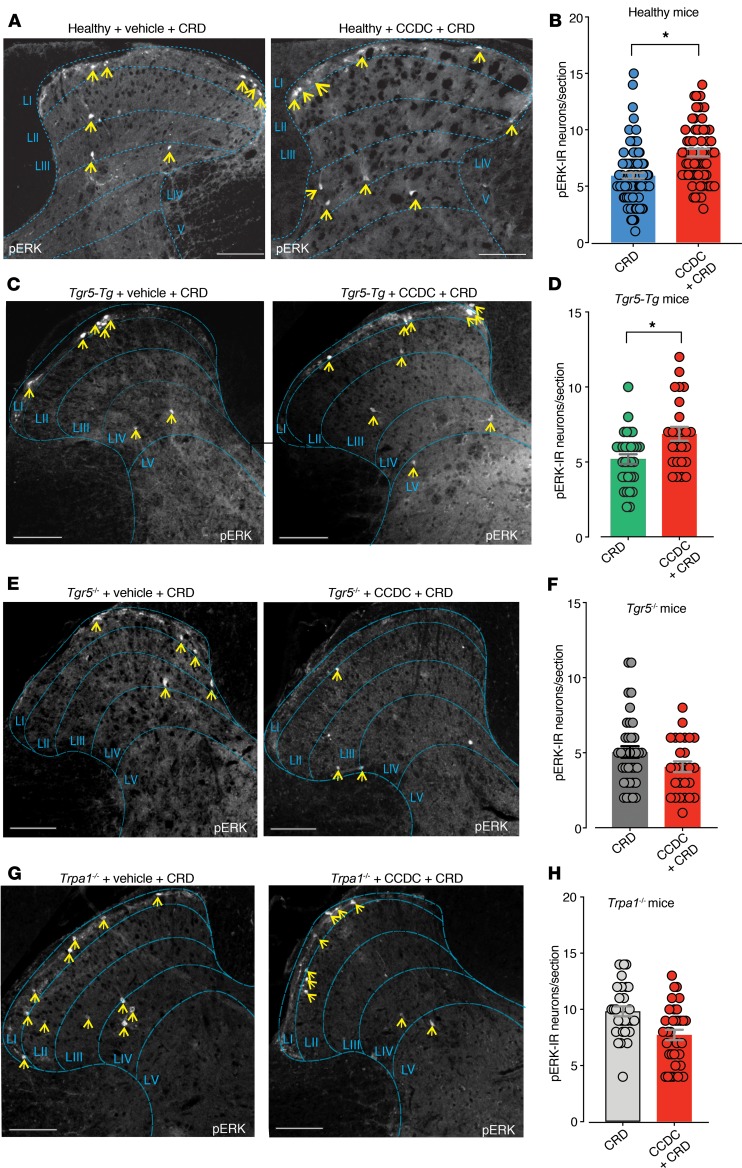
In vivo intracolonic administration of the TGR5 agonist CCDC enhances colorectal distension–induced signaling within the dorsal horn of the spinal cord. (**A**) Colorectal distension (CRD) at a pressure of 40 mmHg in healthy mice results in activation of dorsal horn (DH) neurons within the thoracolumbar (T10-L1) spinal cord, as indicated by phospho–MAP-kinase-ERK-1/2 immunoreactivity (pERK-IR, yellow arrows). pERK-IR neurons within the thoracolumbar DH, activated in response to 40 mmHg CRD, were predominantly located in laminae I–IV. (**B**) Group data showing that mice pretreated with intracolonic CCDC (100 μM) displayed significantly more pERK-IR DH neurons within the thoracolumbar spinal cord following 40 mmHg CRD compared with 40 mmHg CRD alone (*P < 0.05; dots indicate individual counts in spinal cord sections from CRD mice [*N* = 7] vs. CCDC + CRD mice [*N* = 7]). (**C** and **D**) Similarly, intracolonic pretreatment with CCDC in mice overexpressing TGR5 (*Tgr5-Tg*) increased the number of pERK-IR–activated neurons following 40 mmHg CRD, compared with 40 mmHg CRD alone (*P < 0.05; *Tgr5-Tg* CRD mice [*N* = 4] vs. *Tgr5-Tg* CCDC + CRD mice [*N* = 4]). (**E** and **F**) In contrast, intracolonic pretreatment with CCDC in *Tgr5^–/–^* mice did not result in an increase in pERK-IR–activated neurons following 40 mmHg CRD, compared with 40 mmHg CRD alone (*P* > 0.05, *Tgr5^–/–^* CRD mice [*N* = 4] vs.*Tgr5^–/–^* CCDC + CRD mice [*N* = 4]). (**G** and **H**) *Trpa1^–/–^* mice administered an intracolonic pretreatment with CCDC did not display increased numbers of pERK-IR neurons following 40 mmHg CRD, compared with 40 mmHg CRD alone (*P* > 0.05, CRD mice [*N* = 4] vs. CCDC + CRD mice [*N* = 4]). Data presented are mean ± SEM. *P* values determined by unpaired *t* tests (**B**, **D**, **F**, and **H**). Dots represent data from individual sections of spinal cord from *N* = 4–7 mice. Scale bars: 100μm (**A**, **C**, **E**, and **G**).

To determine how activation and sensitization of colonic afferents by TGR5 and Mrgpr agonists results in altered signaling within colonic pathways in vivo, we identified activated DH neurons by phosphorylated MAP-kinase-ERK-1/2 immunoreactivity (pERK-IR) in response to colorectal distension (CRD) (25–32). In healthy vehicle-treated mice, CRD at a pressure of 40 mmHg resulted in activation of DH neurons within the thoracolumbar (T10-L1) regions of the spinal cord (Figure 5, A and B). Pretreatment of healthy mice with the TGR5 agonist CCDC enhanced CRD-evoked activation of DH neurons (Figure 5, A and B). However, intracolonic administration of CCDC alone in healthy mice did not cause activation of DH neurons within the spinal cord (Supplemental Figure 2). Overall, these findings indicate that intracolonic CCDC induced colonic afferent mechanical hypersensitivity in vivo, which translated to increased neuronal activation within the spinal cord. Consistent with this action of TGR5, intracolonic pretreatment with CCDC in mice overexpressing TGR5 (*Tgr5-Tg*) significantly increased the number of pERK-IR neurons following CRD, compared with CRD plus vehicle in *Tgr5-Tg* mice (Figure 5, C and D). In contrast, intracolonic pretreatment with CCDC in *Tgr5^–/–^* mice did not alter the number of pERK-IR neurons following CRD compared with vehicle plus CRD *Tgr5^–/–^* mice (Figure 5, E and F), suggesting that TGR5 does indeed mediate the effects of CCDC. Finally, *Trpa1^–/–^* mice pretreated with intracolonic CCDC followed by CRD displayed no increase in the number of pERK-IR neurons compared with *Trpa1^–/–^* mice with vehicle plus CRD, confirming that TRPA1 is crucial for TGR5-mediated mechanical hypersensitivity (Figure 5, G and H). We also observed that intracolonic administration of CQ resulted in pronounced activation of neurons within the DH of the spinal cord, consistent with in vivo activation of MRGPRA3 in colonic sensory afferent pathways (Supplemental Figure 3).

### In vivo intracolonic administration of pruritogenic agonists increases mechanically evoked responses to CRD and alters animal behavior.

**Figure 6:**
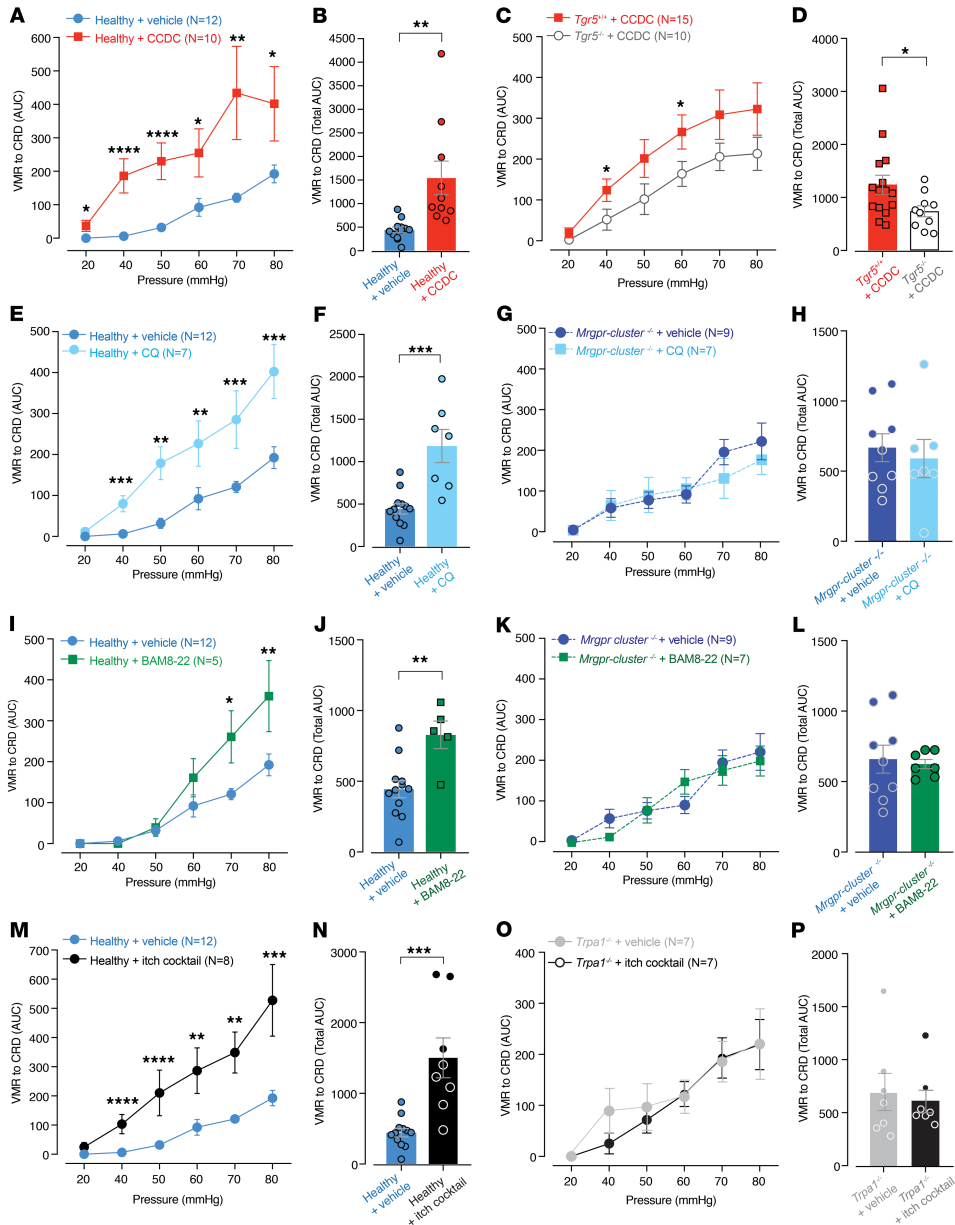
In vivo intracolonic administration of TGR5, MrgprA3, and MrgprC11 agonists, alone or in combination, induces visceral hypersensitivity to colorectal distension. (**A**) Intracolonic administration of CCDC (100μM) resulted in significantly enhanced visceromotor responses (VMRs) to colorectal distension (CRD) in healthy mice, with significant increases observed across all distension pressures. (**B**) Group data expressed as the total AUC of the VMR to CRD shows significantly elevated responses following intracolonic CCDC. Each dot represents the total AUC from an individual animal. (**C**) *Tgr5^–/–^* mice administered intracolonic CCDC (100 μM) showed significantly reduced VMRs compared with *Tgr5*^+/+^ littermates administered intracolonic CCDC. (**D**) Significantly reduced total VMRs in *Tgr5^–/–^* mice administered CCDC compared with *Tgr5*^+/+^. (**E**) Healthy mice administered intracolonic chloroquine (CQ; 10 μM) have significantly elevated VMRs, particularly at 40–80 mmHg distension. (**F**) Intracolonic CQ significantly enhanced total VMRs compared with vehicle. (**G**) *Mrgpr-cluster*^–/–^ mice intracolonically administered 10 μM CQ did not show altered VMRs nor altered (**H**) total VMR relative to *Mrgpr-cluster*^–/–^ mice administered vehicle (*P* > 0.05). (**I**) Mice administered intracolonic BAM8-22 (20 μM) have significantly elevated VMRs, particularly at noxious distension pressures of 70–80 mmHg. (**J**) Intracolonic BAM8-22 significantly enhanced total VMRs compared with vehicle. (**K**) *Mrgpr-cluster*^–/–^ mice intracolonically administered 20 μM BAM8-22 had unaltered VMRs and unaltered (**L**) total VMRs to CRD relative to *Mrgpr-cluster*^–/–^ mice administered vehicle (*P* > 0.05). (**M**) An intracolonic itch cocktail consisting of a combination of CCDC (100 μM), BAM8-22 (20 μM), and CQ (10 μM) significantly enhanced VMRs in healthy mice. This hypersensitivity was evident at 40–50 mmHg, 60–70 mmHg, and 80 mmHg. (**N**) The itch cocktail also significantly enhanced the total VMR compared with vehicle. (**O**) *Trpa1^–/–^* mice intracolonically administered the itch cocktail did not show altered VMRs relative to vehicle-administered *Trpa1*^–/–^ mice (*P* > 0.05). (**P**) Total VMR was unchanged in *Trpa1^–/–^* mice administered the itch cocktail compared with vehicle (*P* > 0.05). Data represent mean ± SEM. *P* values determined by generalized estimating equations, followed by least significant difference post hoc tests (**A**, **C**, **E**, **G**, **I**, **K**, **M**, **O**) or by unpaired *t* tests (**B**, **D**, **F**, **H**, **J**, **L**, **N**, **P**). *P < 0.05, **P < 0.01, ***P < 0.001, ****P < 0.0001.

We next assessed whether TGR5- and Mrgpr-induced activation of colonic afferents resulted in alterations in visceral sensitivity evoked by CRD in vivo. We measured the visceromotor response (VMR) to increasing CRD pressures by recording electromyographic (EMG) activity from electrodes surgically implanted into the abdominal muscles (30, 33–35). In healthy mice, intracolonic administration of CCDC significantly enhanced VMRs to CRD at all distension pressures, indicating visceral hypersensitivity (Figure 6, A and B, and Supplemental Figure 4). In comparison, intracolonic CCDC in *Tgr5^–/–^* mice failed to induce the elevated VMR to CRD observed in *Tgr5*^+/+^ mice administered intracolonic CCDC (Figure 6, C and D, and Supplemental Figure 4). Intracolonic administration of the MrgprA3 agonist CQ significantly enhanced the VMR to CRD in healthy mice, particularly at pressures ≥ 40 mmHg (Figure 6, E and F, and Supplemental Figure 4). However, CQ did not alter the VMR to CRD in *Mrgpr-cluster^–/–^* mice (Figure 6, G and H, and Supplemental Figure 4), confirming the role of Mrgprs in the actions of CQ in colonic pathways. Intracolonic administration of the MrgprC11 agonist BAM8-22 in healthy mice also significantly enhanced the VMR to CRD, although this increase was most apparent at higher, noxious distension pressures of ≥ 60 mmHg (Figure 6, I and J, and Supplemental Figure 4). In contrast, BAM8-22 did not alter the VMR to CRD in *Mrgpr-cluster^–/–^* mice (Figure 6, K and L, and Supplemental Figure 4). Notably, these CCDC-, CQ-, and BAM8-22–induced changes in VMR to CRD were not due to changes in colonic compliance (Supplemental Figure 5, A–F), suggesting that the actions observed occurred via activation of receptors on afferent endings. Overall, these data show that application of the individual agonists for TGR5, MrgprA3, and MrgprC11 can each induce visceral hypersensitivity to CRD in healthy mice.

Since TGR5, MrgprA3, and MrgprC11 are expressed in distinct and overlapping populations of colon-innervating DRG neurons, we determined if coadministration of these agonists, as an “itch cocktail,” also exacerbated visceral hypersensitivity. Concurrent intracolonic administration of CCDC, CQ, and BAM8-22 evoked pronounced increases in the VMR to CRD at all distension pressures and significantly increased the total VMR (Figure 6, M and N, and Supplemental Figure 4). In contrast, *Trpa1^–/–^* mice intracolonically administered the itch cocktail did not show altered VMRs to CRD relative to vehicle-administered *Trpa1^–/–^* mice (Figure 6, O and P, Supplemental Figure 4), confirming that TRPA1 contributes to TGR5-, MrgprA3-, and MrgprC11-induced mechanical hypersensitivity in colonic afferent pathways.

**Figure 7:**
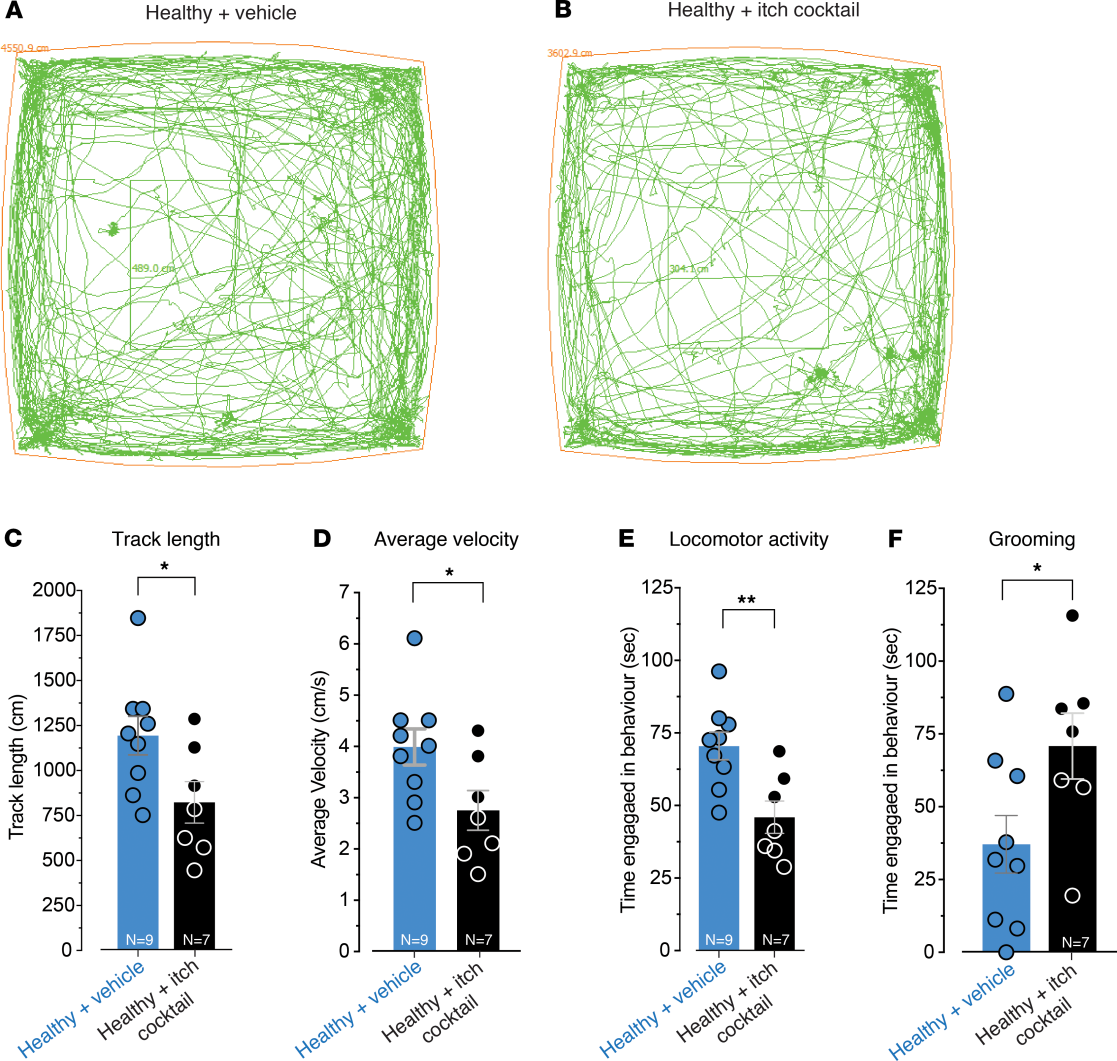
In vivo intracolonic administration of an itch cocktail consisting of TGR5, MrgprA3, and MrgprC11 agonists alters animal behavior. Representative track paths are shown for a (**A**) healthy mouse intracolonically administered vehicle (saline) and for a (**B**) healthy mouse intracolonically administered an itch cocktail consisting of a combination of CCDC (100 μM), BAM8-22 (20 μM), and CQ (10 μM). Intracolonic administration of the itch cocktail significantly reduced (**C**) the total track length covered (*P < 0.05; vehicle, *N* = 9; itch cocktail, *N* = 7), (**D**) the average velocity of travel (*P < 0.05; vehicle, *N* = 9; itch cocktail, *N* = 7), and (**E**) locomotor activity time compared with vehicle treatment (**P < 0.01; vehicle, *N* = 9; itch cocktail, *N* = 7). (**F**) Intracolonic administration of the itch cocktail also significantly increased grooming behavior compared with vehicle (*P < 0.05; vehicle, *N* = 9; itch cocktail, *N* = 7). Data represent mean ± SEM. Dots represent values from individual mice. *P* values were by unpaired *t* tests (**C**, **D**, **E**, **F**).

To determine if concurrent activation of TGR5, MrgprA3, and MrgprC11 has effects beyond mechanically evoked visceral sensitization, we also recorded animal behavior in response to intracolonic administration of the itch cocktail. Healthy mice coadministered CCDC, CQ, and BAM8-22 covered significantly less distance in their enclosure (Figure 7, A–C), had a slower average velocity of travel (Figure 7D), displayed reduced locomotor activity (Figure 7E), and displayed more grooming behavior (Figure 7F) compared with vehicle-administered mice. These behavioral changes were not observed when TGR5, MrgprA3, or MrgprC11 agonists were applied individually (Supplemental Figure 6), suggesting full recruitment of these irritant pathways is required to induce profound behavioral changes in these mice. Notably, mice intracolonically administered CCDC, CQ, or BAM8-22, either individually or in combination, did not display increased scratching behavior (Supplemental Figure 7), suggesting that the agonists were localized to the colon and did not reach the systemic circulation. Overall, our results demonstrate crucial individual and combined roles for TGR5, MrgprA3, and MrgprC11 in the sensitization of colonic afferent pathways and the resultant changes in spinal cord processing, responsiveness to CRD, and animal behavior.

### TGR5, MrgprA3, and MrgprC11 also contribute to the sensitization of colonic afferent pathways during CVH.

In order to determine if the roles of TGR5, MrgprA3, and MrgprC11 in evoking visceral hypersensitivity extends into disease states, we used a CVH mouse model of IBS. CVH was induced by administration of intracolonic trinitrobenzenesulphonic acid (TNBS), which has been shown to induce colitis (36, 37). While colonic inflammation spontaneously heals over a 7-day period, these mice subsequently develop chronic mechanical hypersensitivity of colonic afferents in the postinflammatory state (25–27, 30, 34, 36, 38), display neuroplasticity within spinal cord pathways (16, 30, 31) and exhibit visceral hypersensitivity to CRD (30, 34).

**Figure 8:**
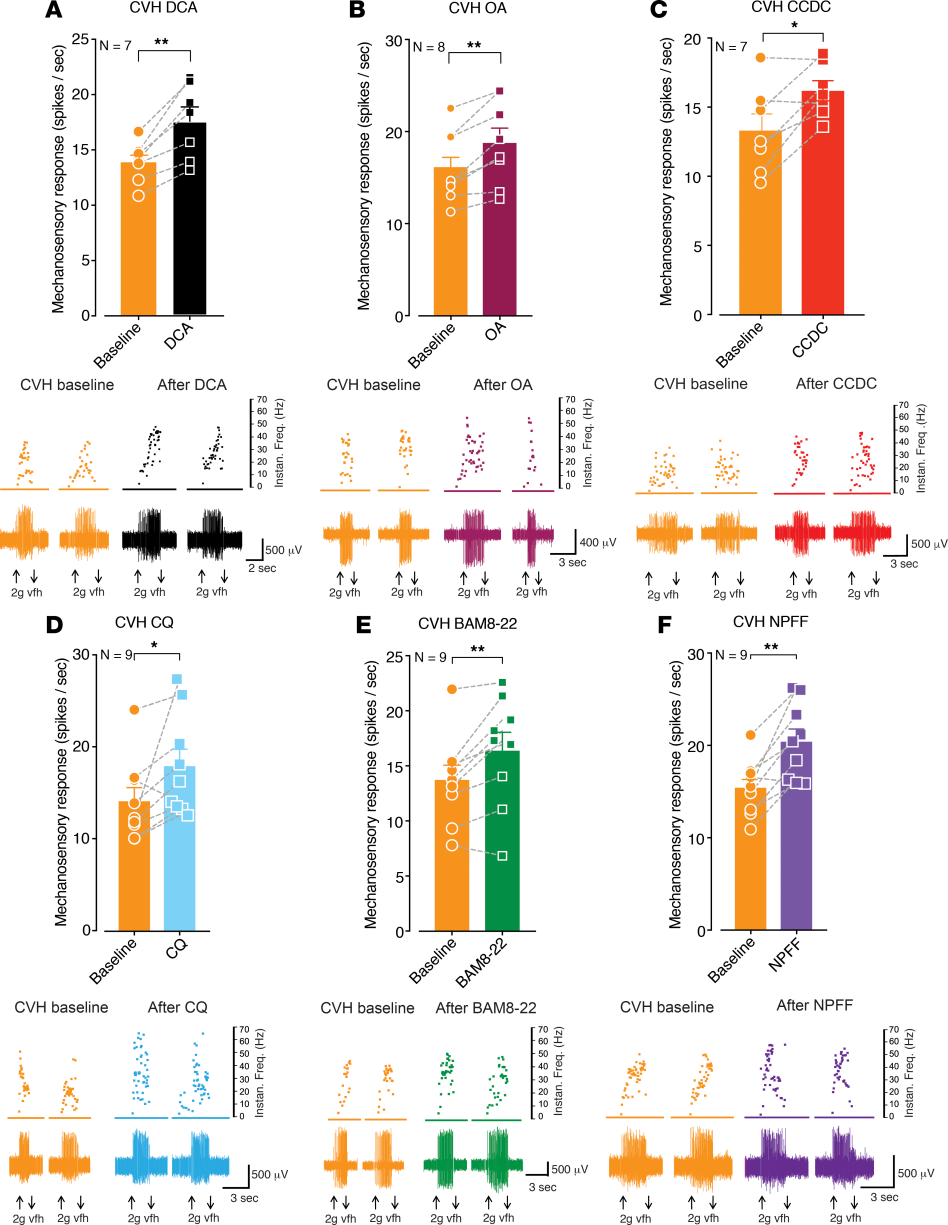
TGR5, MrgprA3, and MrgprC11 agonists evoke mechanical hypersensitivity in colonic afferents from mice with chronic visceral hypersensitivity (CVH). (**A**) Application of the TGR5 agonist deoxycholic acid (DCA; 100 μM) to the colonic mucosa of CVH mice induces mechanical hypersensitivity of colonic nociceptors (**P < 0.01, *N* = 7). Dots represent values from individual CVH afferents before and after DCA application. Lower panels show representative recordings from a single colonic afferent nerve fiber from a CVH mouse responding to a 2 g vfh before and after incubation with DCA. (**B**) Application of the TGR5 agonist oleanolic acid (OA; 100 μM for 5 minutes) also caused mechanical hypersensitivity in nociceptors from CVH mice (**P < 0.01, *N* = 8). (**C**) The TGR5 agonist CCDC (100 μM for 5 minutes) also evoked mechanical hypersensitivity of colonic nociceptors from CVH mice (*P < 0.05, *N* = 7). (**D**) Colonic nociceptors from CVH mice also displayed mechanical hypersensitivity to the application of the MrgprA3 agonist chloroquine (CQ, 10 μM for 5 minutes, *P < 0.05, *N* = 9), (**E**) MrgprC11 agonist BAM8-22 (20 μM for 5 minutes, **P < 0.01, *N* = 9), and (**F**) the combined MrgprC11/MrgprA4 agonist neuropeptide FF (NPFF; 5 μM for 5 minutes, **P < 0.01, *N* = 9 mice). Data represent Mean ± SEM. *P* values determined by paired *t* tests (**A–F**).

**Figure 9:**
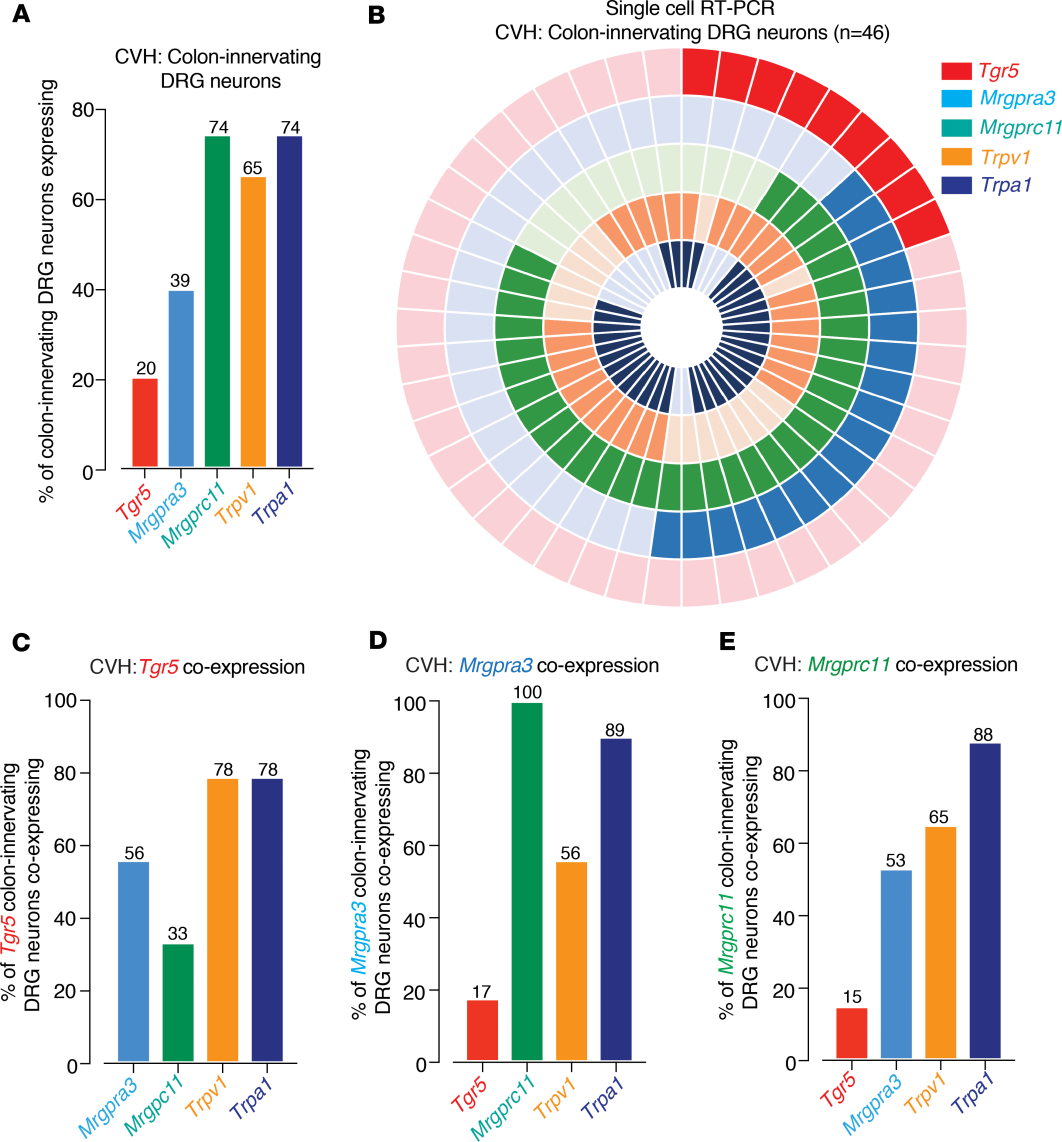
Distinct and overlapping expression patterns for *Tgr5*, *Mrgpra3,* and *Mrgprc11* in colon-innervating DRG neurons from CVH mice. (**A**) Single-cell RT-PCR of 46 retrogradely traced colon-innervating DRG neurons from 4 CVH mice reveals that subpopulations express *Tgr5* (20%), *Mrgpra3* (39%), *Mrgprc11* (74%), *Trpv1* (65%), and *Trpa1* (74%). (**B**) Donut plot showing expression and coexpression of genes encoding *Tgr5*, *Mrgpra3*, *Mrgprc11*, *Trpv1*, and *Trpa1* in 46 individual retrogradely traced colon-innervating DRG neurons from CVH mice. Each color represents an individual gene, with expression marked by bold shading (*Tgr5,* outer ring; *Trpa1*, inner ring). Some CVH DRG neurons express all targets, while other neurons express combinations of targets. (**C–E**) Group data showing (**C**) *Tgr5*, (**D**) *Mrgpra3*, and (**E**) *Mrgprc11* are expressed individually within subpopulations of colon-innervating DRG neurons and also coexpressed together in other subpopulations. For example, of the *Tgr5* expressing colon-innervating DRG neurons from CVH mice, 56% coexpress *Mrgpra3*, and 33% coexpress *Mrgprc11*. *Tgr5*, *Mrgpra3*, and *Mrgprc11* also heavily coexpress with *Trpv1* and, in particular, *Trpa1* in CVH states.

Colonic afferents from CVH mice displayed mechanical hypersensitivity relative to afferents from healthy mice (Supplemental Figure 8), as described previously (25–27, 30, 34, 36, 38). Interestingly, application of the TGR5 agonists DCA, OA, or CCDC further amplified mechanical hypersensitivity in CVH colonic afferents, above their already-elevated baseline levels (Figure 8, A–C). We also observed that a subpopulation of CVH afferents displayed action potential firing to application of the TGR5 agonists in the absence of mechanical stimuli, which rarely occurred in healthy colonic afferents (Supplemental Figure 9). Individual application of the MrgprA3 agonist CQ, the MrgprC11 agonist BAM8-22, or the MrgprC11/MrgprA4 agonist NPFF also further amplified mechanical hypersensitivity in CVH colonic afferents (Figure 8, D–F). This was also associated with action potential firing to application of the individual Mrgpr agonists, which rarely occurred in healthy colonic afferents (Supplemental Figure 9). Single-cell RT-PCR from CVH mice showed that 20% of colon-innervating DRG neurons expressed *Tgr5*, while 39% expressed *Mrgpra3* and 74% expressed *Mrgprc11*, with 65% expressing *Trpv1* and 74% *Trpa1* (Figure 9, A and B). Compared with healthy colon-innervating DRG neurons, this represented a significant increase in the proportion of DRG neurons expressing *Mrgprc11* or *Trpa1* in CVH states (Supplemental Figure 10). There were also significant changes in the coexpression profiles of CVH colon-innervating DRG neurons (Figure 9, C–E), with significantly more *Mrgpra3* expressing CVH DRG neurons coexpressing *Mrgprc11* and *Trpa1* (Supplemental Figure 10) and significantly fewer *Mrgprc11* neurons coexpressing *Trpv1* (Supplemental Figure 10).

**Figure 10:**
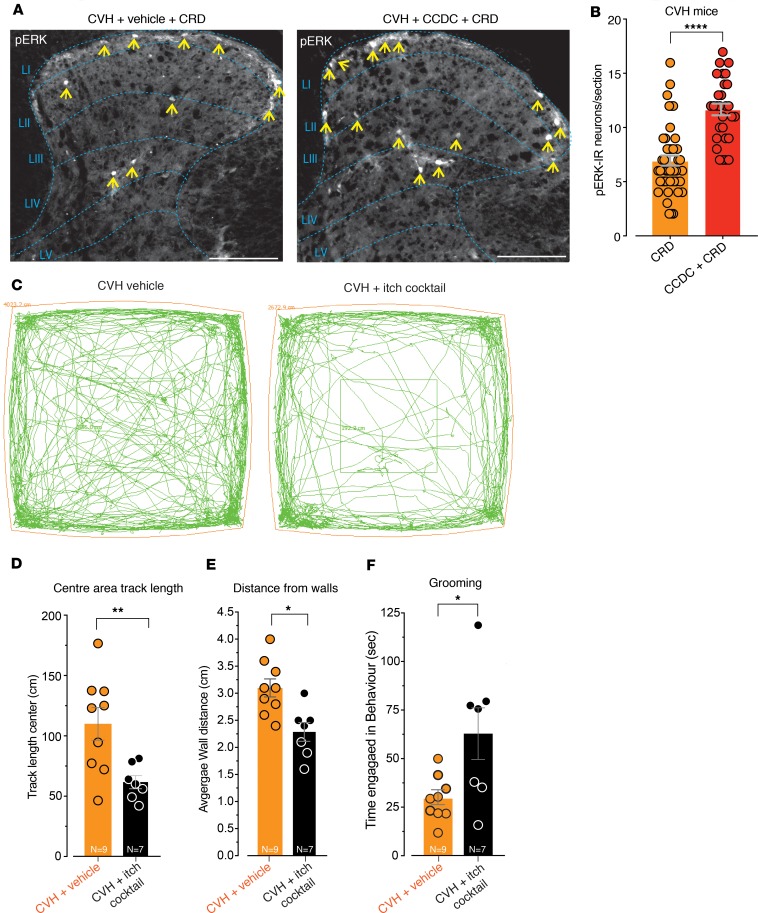
In vivo intracolonic administration of pruritogenic agonists in CVH mice increases dorsal horn neuron activation in response to colorectal distension and alters animal behavior. (**A**) CRD at a pressure of 40 mmHg in CVH mice results in activation of DH neurons within the thoracolumbar (T10-L1) spinal cord, as indicated by pERK-IR (yellow arrows). CVH mice pretreated with intracolonic CCDC (100 μM) display more DH neurons in the spinal cord following 40 mmHg CRD. Scale bars: 100 μm. (**B**) Group data showing that CVH mice pretreated with intracolonic CCDC (100 μM) displayed significantly more pERK-IR DH neurons within the spinal cord following 40 mmHg CRD compared with 40 mmHg CRD alone (****P < 0.0001, dots indicate individual counts in spinal cord sections from *N* = 6 CVH CRD and *N* = 6 CVH CCDC+CRD). (**C**) Representative track paths are shown for individual CVH mice administered either intracolonic vehicle (saline) or an itch cocktail consisting of a combination of CCDC (100 μM), BAM8-22 (20 μM), and CQ (10 μM). (**D–F**) Intracolonic administration of the itch cocktail to CVH mice significantly reduces (**D**) their track length covered within the central area of the observation enclosure (**P < 0.01; CVH + vehicle, *N* = 9; CVH + itch cocktail, *N* = 7) and (**E**) reduces their distance from the walls of the enclosure (*P < 0.05; CVH + vehicle, *N* = 9; CVH + itch cocktail, *N* = 7) compared with vehicle-treated CVH mice. (**F**) Intracolonic administration of the itch cocktail to CVH mice also significantly increased grooming behavior compared with CVH vehicle-treated mice (*P < 0.05; CVH + vehicle, *N* = 9; CVH + itch cocktail, *N* = 7). Data represent mean ± SEM. Dots represent values from individual mice. *P* values determined by unpaired *t* tests (**B**, **D**, **E**, **F**).

We also found that intracolonic administration of CCDC alone in CVH mice resulted in pERK-IR within DH neurons of the spinal cord (Supplemental Figure 2). Furthermore, CVH mice pretreated with CCDC displayed significantly more pERK-IR DH neurons within the spinal cord following 40 mmHg CRD compared with CVH mice with vehicle plus CRD (Figure 10, A and B). These findings indicate that in vivo intracolonic CCDC activates colonic afferents and also induces mechanical hypersensitivity in CVH mice. In terms of behavioral responses, CVH mice intracolonically administered the itch cocktail of concurrent CCDC, CQ, and BAM8-22 displayed significantly reduced movement in terms of the distance travelled within the central observational area of the enclosure (Figure 10, C and D), a significantly decreased distance from the walls of the enclosure (Figure 10E), and a significantly increased time spent grooming (Figure 10F). However, these CVH mice did not display increased scratching behavior in response to the intracolonic itch cocktail (Supplemental Figure 7). Overall, our results demonstrate that TGR5, MrgprA3, and MrgprC11 each contribute to the sensitization of colonic afferent pathways in CVH states. There is an increase in MrGprC11- and TRPA1-dependent mechanisms in CVH and that agonists for TGR5, MrgprA3, and MrgprC11 profoundly alter the behavior of CVH mice.

### Human DRG neurons express TGR5 and MrgprX1 and respond to pruritogenic agonists.

**Figure 11:**
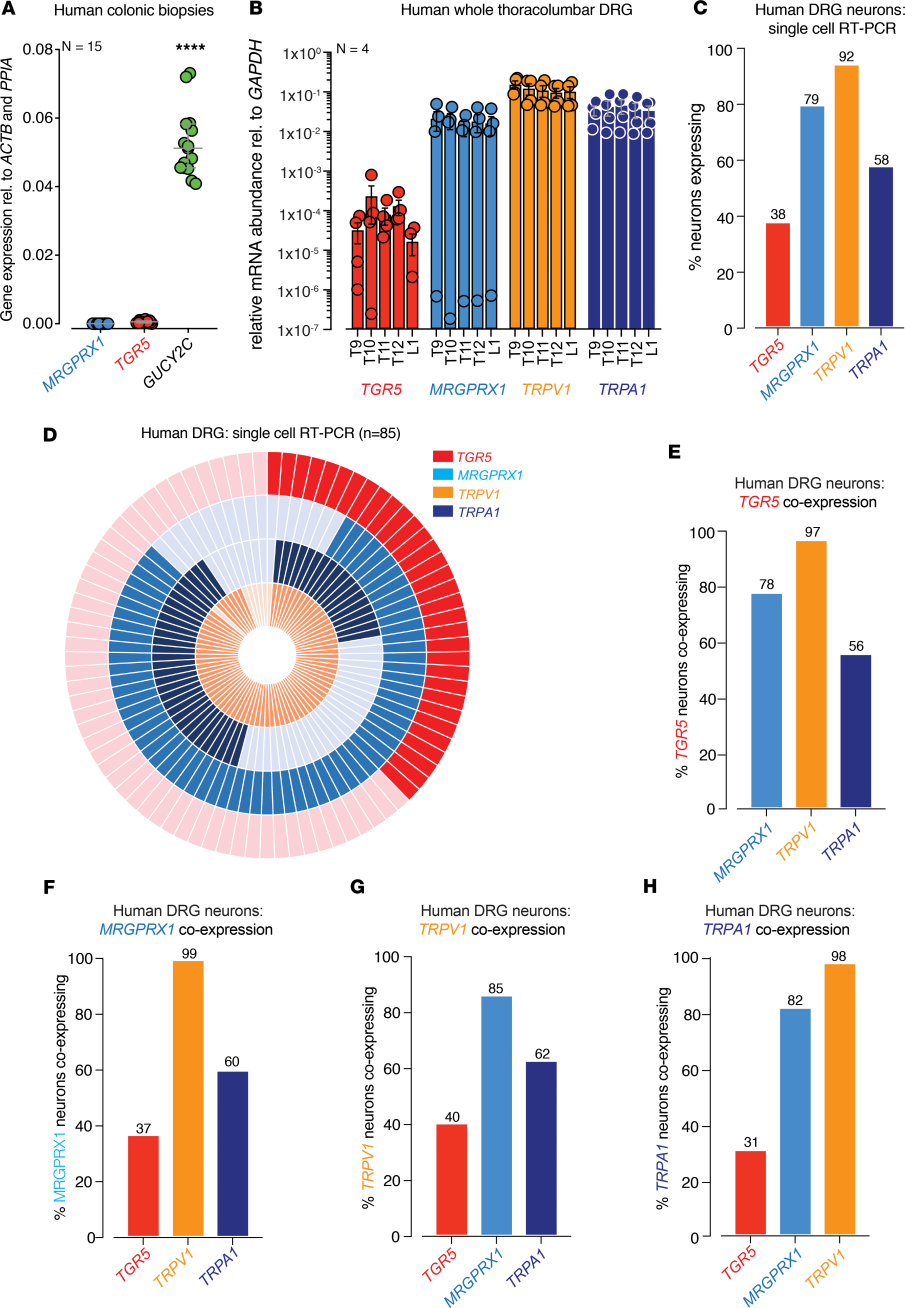
Human DRG neurons coexpress *TRG5*, *MrgprX1*, *Trpv1*, and *Trpa1*. (**A**) qPCR analysis from colonic biopsies from healthy human subjects show low levels of mRNA expression for *Tgr5* and absent *MrgprX1* (human ortholog of the mouse *Mrgpra3* and *Mrgprc11*) compared with a known epithelial target *gucy2c* (GC-C, ****P < 0.0001, *N* = 15 subjects. Dots represent averaged values from each patient sample). (**B**) qPCR expression analysis of whole human thoracolumbar (TL; T9-L1) DRG from 4 human donors. Analysis reveals abundant expression of *MrgprX1*, *Trpv1*, and *Trpa1*, plus expression of the bile acid receptor *Tgr5*. Dots represent averaged values from each donor at each DRG level. (**C**) Single-cell RT-PCR analysis showing the percentage of individual human DRG neurons expressing the *Tgr5*, *MrgprX1*, *Trpv1*, and *Trpa1*. Data show that, of the 85 individual human thoracolumbar DRG neurons examined, 38% express *Tgr5*, 79% express *MrgprX1*, 92% expressed *Trpv1*, with 58% expressing *Trpa1*. (**D**) Donut plot analysis showing coexpression profiles of 85 individual human TL DRG neurons using single-cell RT-PCR for *Tgr5*, *MrgprX1*, *Trpv1*, and *Trpa1*. (**E**) Of the 38% of human TL DRG neurons expressing *Tgr5*, 78% coexpress *MrgprX1*, 97% coexpress *Trpv1*, with 56% coexpressing *Trpa1*. (**F**) Of the 79% of human DRG neurons expressing *MrgprX1*, 37% coexpress *Tgr5*, 99% coexpress *Trpv1*, with 60% coexpressing *Trpa1*. (**G**) *Trpv1-*expressing human DRG neurons also express *Tgr5* (40%), *MrgprX1* (85%), and *Trpa1* (62%). (**H**) *Trpa1*-expressing human DRG neurons also express *Tgr5* (31%), *MrgprX1* (82%), and *Trpv1* (98%). Data in **A** and **B** represent mean ± SEM. *P* values determined by 1-way ANOVA with Tukey’s multiple comparison tests (**A**).

To further investigate the translatability of our findings, we determined the mRNA expression profiles of *TGR5* and *MrgprX1* (the human ortholog of murine *Mrgpra3* and *Mrgprc11*) in human tissue and also tested the responsiveness of human DRG neurons to TGR5, MrgprX1, TRPV1, and TRPA1 agonists. Firstly, using colonic biopsies from 15 human healthy subjects, we found that *TGR5* had low expression compared with a known epithelial target *GUCY2C,* while *MrgprX1* was absent (Figure 11A), which is consistent with our findings in mouse colonic mucosa (Figure 3A). qPCR of T9-L1 whole thoracolumbar DRG from 4 human donors showed expression of *TGR5*, with greater abundance of *MrgprX1* and, in particular, *TRPA1* and *TRPV1* (Figure 11B). Single-cell RT-PCR from 85 individual human DRG neurons, of predominately smaller diameter, demonstrated that 38% expressed *TGR5*, 79% expressed *MrgprX1*, 92% expressed *TRPV1*, and 58% expressed *TRPA1* (Figure 11, C and D). Consistent with our observations from mouse DRG, we found that *TGR5* and *MrgprX1* were expressed in both distinct and overlapping populations of human DRG neurons, which heavily coexpressed *TRPV1* or *TRPA1* (Figure 11D). Specifically, of the *TGR5*-expressing human DRG neurons, 78% coexpressed *MrgprX1*, 97% coexpressed *TRPV1*, and 56% coexpressed *TRPA1* (Figure 11E). Of the *MrgprX1*-expressing population, 37% coexpressed *TGR5*, 99% coexpressed *TRPV1*, and 60% coexpressed *TRPA1* (Figure 11F). Of the *TRPV1*-expressing population, 40% coexpressed *TGR5*, 85% coexpressed *MrgprX1,* and 62% coexpressed *TRPA1* (Figure 11G), while — of the *TRPA1*-expressing population — 31% coexpressed *TGR5*, 82% coexpressed *MrgprX1*, and 98% coexpressed *TRPV1* (Figure 11H).

**Figure 12:**
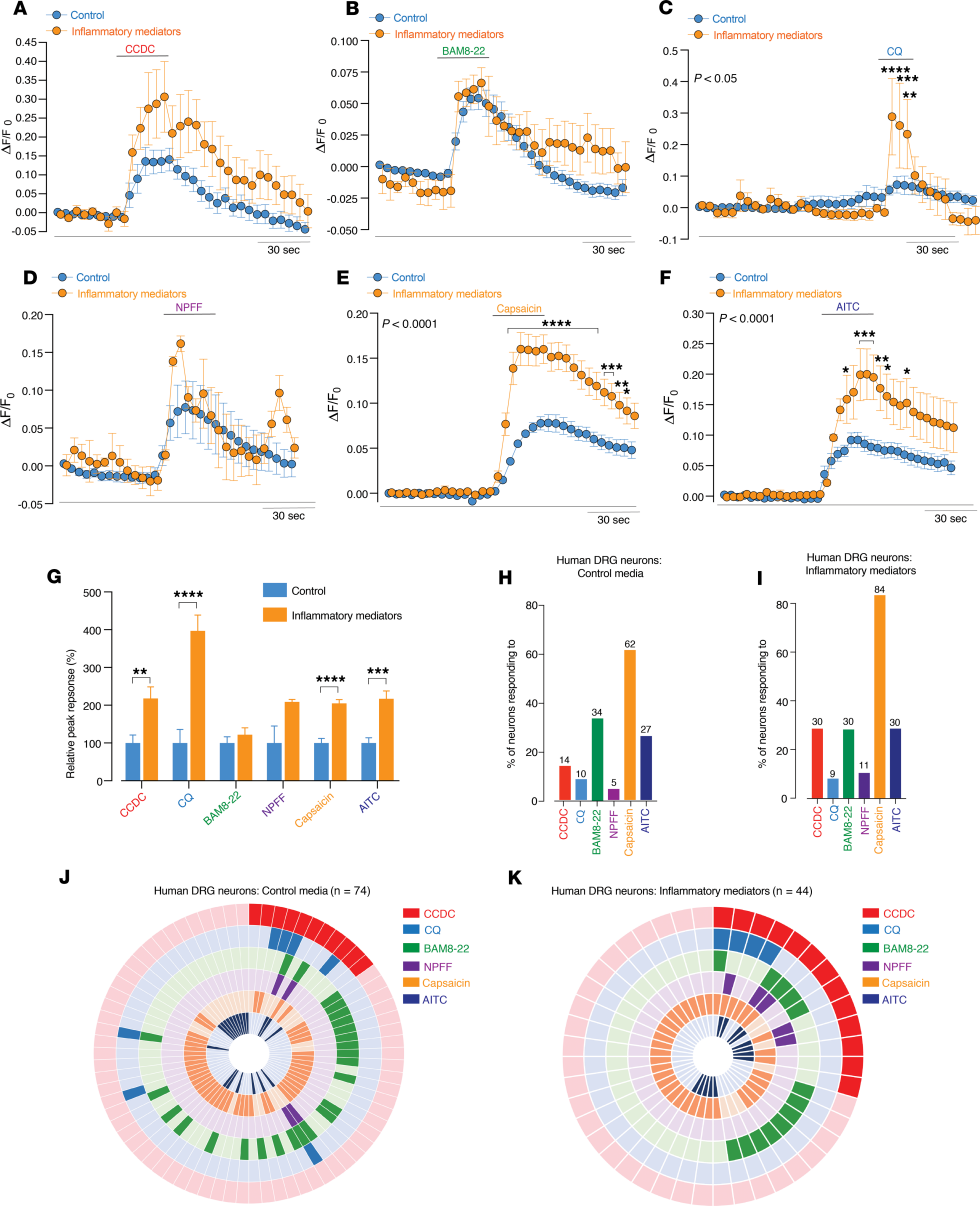
Human DRG neurons respond to pruritogenic agonists for TGR5, in addition to the MrgprX1 agonists chloroquine and BAM8–22. (**A–F**) Human DRG neurons were cultured in control media, and in order to simulate a pathological state, a subset of cultures incubated with inflammatory mediators. This consisted of histamine (10 μM), PGE II (10 μM), serotonin (10 μM), and bradykinin (10 μM) being incubated with the neurons for 2 hours before Ca^2+^ imaging experiments commenced. Human DRG neurons from this cohort are referred to as inflammatory mediators. Grouped data of Ca^2+^ responses in control (*n* = 74) and inflammatory mediator (*n* = 44) cultured human DRG neurons to application of the (**A**) TGR5 agonist CCDC (100 µM), MrgprX1 agonists (**B**) BAM8-22 (2 μM), (**C**) CQ (1 μM), (**D**) NPFF (2 μM), (**E**) TRPV1 agonist capsaicin (100 nM), and (**F**) TRPA1 agonist AITC (50 M). Two-way ANOVA indicate responses to CQ (*P < 0.05), capsaicin (***P < 0.001), and AITC (**P < 0.01) are all significantly increased in neurons that had been exposed to inflammatory mediators. (**G**) Peak response of neurons to CCDC (**P < 0.01), CQ (****P < 0.0001), capsaicin (****P < 0.0001), and AITC (***P < 0.001) were all significantly increased in human DRG neurons incubated with inflammatory mediators. (**H** and **I**) Human DRG neurons from (**H**) control and (**I**) inflammatory mediator cultures responding to CCDC, CQ, BAM8-22, NPFF, capsaicin, and AITC. (**J** and **K**) Donut plot analysis showing the functional coexpression profiles as determined by Ca^2+^ imaging of (**J**) 74 individual human DRG neurons from control cultures and (**K**) 32 individual human DRG neurons from inflammatory mediator cultures in response to CCDC, CQ, BAM8-22, NPFF, capsaicin, and AITC. Data presented are mean ± SEM. *P* values determined by 2-way ANOVA and Bonferroni post hoc tests (significance indicated within panels) (**A–F**) or unpaired *t* tests (**G**).

Using Ca^2+^ imaging of dissociated and cultured human DRG neurons, we found that subpopulations of neurons were activated by the application of CCDC (14%; Figure 12, A, G, and H), BAM8-22 (34%; Figure 12, B, G, and H), CQ (10%; Figure 12, C, G, and H), and NPFF (5%; Figure 12, D, G, and H), as indicated by robust increases in [Ca^2+^]_i_ (Figure 12, A–D). Many of these neurons also responded to capsaicin (62%; Figure 12, E, G, and H) or AITC (27%; Figure 12, F, G, and H). In order to simulate a pathological state, we transiently incubated neurons in culture with inflammatory mediators (histamine, PGE II, serotonin, bradykinin) for 2 hours prior to the Ca^2+^ imaging experiments. Human DRG neurons from these cultures displayed greater amplitudes of response to the application of CCDC (Figure 12, A and G), CQ (Figure 12, C and G), capsaicin (Figure 12, E and G), and AITC (Figure 12, F and G). Overall, 30% of neurons from the inflammatory mediator cultures responded to CCDC, 9% to CQ, 30% to BAM8-22, and 11% to NPFF, with 84% responding to capsaicin and 30% to AITC (Figure 12I). Overall, significantly more neurons from the inflammatory mediator cultures responded to capsaicin than in the normal untreated cultures (Figure 12, H–K, and Supplemental Figure 11). Overall, these findings in human DRG neurons largely resemble our findings in mouse colon-innervating DRG neurons and suggest that TGR5 and MrgprX1 play important roles in pruritogenic signaling from human DRG neurons in a variety of conditions.

## Discussion

IBS affects ~11% of the global population, and therapeutic treatments are currently lacking (15). Persistent hypersensitivity of sensory pathways innervating the colon is linked to the initiation, development, and maintenance of chronic discomfort and abdominal pain in IBS patients (15, 16, 39). Therefore, determining the mechanisms contributing to these processes is crucial. In the current study, we show that activation of TGR5, MrgprA3, or MrgprC11, commonly considered as itch receptors, either individually or collectively cause fundamental signaling changes within colonic afferent pathways in healthy states. Crucially, we also show that these mechanisms persist and, in the case of MrgprC11, are augmented in CVH states. Therefore, this study provides insights on how the activation of pruritogenic receptors initiates colonic hypersensitivity and, importantly, how these receptors contribute to chronic hypersensitivity. Accordingly, this information may afford novel therapeutic strategies by directly targeting these receptors for the treatment of chronic discomfort and abdominal pain in IBS.

In the current study, we found that mRNA for the pruritogenic receptors *Tgr5*, *Mrgpra3*, and *Mrgprc11* were all expressed in a remarkably large population (19%, 27%, and 40%, respectively) of mouse colon-innervating DRG neurons in healthy states. Correspondingly, agonists for MrgprA3 (CQ), MrgprC11 (BAM8-22), and TGR5 (DCA, TLCA, CCDC) activated ~20%–35% of isolated colon-innervating DRG neurons from healthy mice. Moreover, the individual agonists for MrgprA3, MrgprC11, or TGR5 each induced mechanical hypersensitivity in subpopulations of colonic afferents from healthy mice. The ex vivo and in vivo sensitizing effects of CCDC were exacerbated in *Tgr5-Tg*–overexpressing mice and lost in *Tgr5^–/–^* mice, thereby confirming the role of TGR5 in these processes. Furthermore, mechanical hypersensitivity induced by either CQ or BAM8-22 was lost in *Mrgpr-cluster^–/–^* mice, confirming the roles of Mrgprs in this process. In vivo activation of either TGR5, MrgprA3, or MrgprC11 caused pronounced visceral hypersensitivity to CRD. These findings demonstrate clear and crucial individual roles for MrgprA3, MrgprC11, and TGR5 in activating colonic afferent neurons and inducing mechanical hypersensitivity.

The sensitizing effects of TGR5, MrgprA3, or MrgprC11 agonists on colonic afferents likely occurs via neuronal mechanisms. This is because MRGPRA3 and MRGPRC11 (40) are absent from colonic tissues but are expressed on mouse and human DRG neurons. While TGR5 is expressed on colonic afferents, it is also expressed on colonic epithelial cells and on enteric neurons (41, 42). However, we did not observe any changes in muscle compliance in our studies, suggesting the actions we observed were via direct actions on afferents rather than via secondary mechanisms. Indeed, very recent findings show that bile acid sensitize afferents in the proximal colon via 5-HT_3_–dependent mechanisms, while these actions are 5-HT_3_ independent more distally (43). Although not specifically investigated in the current study, TGR5 activation stimulates release of gastrin-releasing peptide (GRP) within the spinal cord (6), while MRGPR activation results in the release of both GRP (44) and natriuretic polypeptide B (4) within the spinal cord to induce scratching (45). These mechanisms may also contribute to the transmission of visceral irritant signaling from the periphery to the spinal cord and is subject to further investigation.

Importantly, we show for the first time to our knowledge that Mrgprs and TGR5 are expressed in both distinct and overlapping populations of neurons. Our single-cell RT-PCR analysis reveals that 62% of colon-innervating DRG neurons from healthy mice express at least 1 of the *Tgr5*, *Mrgprc11* or *Mrgpra3* receptors. This is an important finding, as these different molecular and functional expression profiles would therefore allow individual, overlapping, and additive signals to occur in response to a variety of pruritogenic irritants. To test this in vivo, we administered CCDC, CQ, or BAM8-22 individually to activate either TGR5, MrgprA3, or MrgprC11 on colonic afferents, respectively. In each scenario, mechanical hypersensitivity was evident in response to CRD, with CQ and CCDC evoking visceral hypersensitivity across a wide range of distension pressures. In the case of BAM8-22, visceral hypersensitivity to CRD was observed at more noxious distension pressures. This is consistent with very recent findings showing that BAM8-22 evoked elevated pain responses to CRD in healthy mice (40). While intracolonic administration of the individual agonists for TGR5, MrgprA3, and MrgprC11 evoked hypersensitivity to CRD, they did not fundamentally affect spontaneous animal behavior. When we administered an itch cocktail, consisting of a combination of CCDC, CQ, and BAM8-22, to concurrently activate TGR5, MrgprA3, and MrgprC11 on colonic afferents, this resulted in pronounced mechanical hypersensitivity to CRD across a wide range of distension pressures. Moreover, by recruiting the full complement of afferents within these irritant pathways, we also observed profound changes in spontaneous animal behavior evoked by visceral hypersensitivity, evident by a reduction in locomotor activity and increased grooming.

We found that the itch cocktail–induced mechanical hypersensitivity to CRD in vivo was not evoked in *Trpa1*^–/–^ mice. Also, we did not observe afferent hypersensitivity, nor increased numbers of pERK-IR in the DH of the spinal cord in response to CCDC and CRD in *Trpa1*^–/–^ mice. These results are consistent with the coupling mechanisms described in the skin, whereby TRPA1 has been identified as the downstream target of TGR5 (10), and both MrgprA3 and MrgprC11 (9). These previous studies demonstrated that neither TGR5 (10), MRGPRA3, nor MRGPRC11 (9) agonists directly activate TRPA1. However, *Trpa1*^–/–^ mice display little to no scratching in response to CQ and BAM8-22 (9). Interestingly, the functional coupling between MrgprA3 and TRPA1 is attenuated by disrupting Gβγ intracellular signaling, while coupling between MrgprC11 and TRPA1 requires phospholipase-C (PLC) signaling (9). Similarly, TGR5 also activates TRPA1 to induce itch in mice, with TGR5 activating and sensitizing TRPA1 via a Gβγ- and protein kinase C–mediated (PKC-mediated) mechanisms (10). Although previous studies identify high coexpression of TRPV1 with MrgprA3 and MrgprC11 (9), as also shown in the current study, there appears to be little to no interaction between these targets. CQ- and BAM8-22–evoked Ca^2+^ signaling and neuronal sensitization is profoundly diminished in neurons from *Trpa1*^–/–^ but not *Trpv1*^–/–^ mice (9). Although *Trpv1* coexpresses with *Tgr5*, deletion or antagonism of TRPV1 has no effect on TGR5-induced itch (10). Comparably, in the current study, although we observed *Trpv1* coexpression in *Tgr5-* (78%), *Mrgpra3-* (69%), or *Mrgprc11-*expressing (90%) colon-innervating DRG neurons, mechanical hypersensitivity was completely lost in studies using *Trpa1^–/–^* mice. Accordingly, colonic afferents, like cutaneous afferents, appear to utilize coupling between TGR5, MrgprA3, or MrgprC11 via TRPA1 in order to mediate their sensitizing actions. These findings further highlight TRPA1 as a crucial integrator of sensory signals in colonic afferents by inducing mechanical hypersensitivity in response to bradykinin (22), TNF-α (23), and proteases (46) and now to bile acids, CQ, and BAM8-22. Conversely, histamine-dependent mechanisms in the colon contribute to afferent sensitization via TRPV1-dependent (12) and TRPV4-dependent (47) mechanisms, potentially suggesting divergent mechanisms between histamine-dependent and histamine-independent afferent sensitization.

Our observations raise the question of why functional itch receptors are found in colonic sensory pathways. There are several possible roles for such irritant-sensing pathways in the colon. Firstly, bile acids are normally present in the colonic lumen; they are secreted into the intestinal lumen during feeding, are absorbed in the ileum, and are modified by the colonic microbiome (48). Also, TGR5 in enteric neurons of the colon contributes to bile acid–dependent stimulation of peristalsis (41). Secondly, BAM8-22 is a proteolytically cleaved product of proenkephalin A, an endogenous ligand found throughout peripheral tissues, including the gastrointestinal tract (49, 50). Thirdly, while a well-recognized side-effect of the use of CQ in the treatment of malaria is itch, less-recognized symptoms of CQ treatment include abdominal cramping and pain (14). Therefore, while itch induces protective scratching that removes harmful irritants from the skin, identification of TGR5, *MRGRPA3*, and *MRGRPC11* in colonic afferents may represent an analogous system in the viscera. This would provide protective mechanisms for detecting harmful irritants within the colon and ultimately expel them from the body via activation of sensory afferents and recruitment of defecatory mechanisms (41). Accordingly, increased levels of bile acids are implicated in diarrhea-predominant IBS (51). Based on our current findings, bile acids also contribute to visceral hypersensitivity and the development of abdominal discomfort and pain via activation of TGR5 expressed on colonic afferents. In keeping with such a role, in vivo intracolonic administration of CCDC evoked mechanical hypersensitivity and increased the number of activated neurons within the DH of the spinal cord following CRD. Similarly, in vivo intracolonic CQ administration resulted in the subsequent activation of DH neurons within the spinal cord and evoked mechanical hypersensitivity to CRD.

We also demonstrate that TGR5-, MRGPRA3-, and MRGPRC11-dependent mechanisms extend beyond sensitization of colonic pathways in healthy states. Crucially, by using a CVH model, we show that colonic afferents from CVH mice display mechanical hypersensitivity compared with afferents from healthy mice. Application of CCDC, CQ, or BAM8-22 further enhanced CVH afferent responses to mechanical stimuli, significantly increasing responses above their already-elevated levels. Thus, activation of TGR5, MRGPRA3, or MRGPRC11 in CVH states can further exacerbate visceral hypersensitivity, leading to hyperalgesia. Correspondingly, we also show that afferents from CVH mice were more likely to fire action potentials in response to pruritogens and displayed increased numbers of pERK-IR DH neurons in response to intracolonic CCDC application in the absence of CRD. Notably, significantly more colon-innervating DRG neurons from CVH mice express *Mrgprc11* and *Trpa1*, with a significant increase in the proportion of *Mrgpra3*-expressing neurons now also coexpressing *Mrgprc11* and *Trpa1*. Our single-cell RT-PCR analysis reveals that 83% of colon-innervating DRG neurons from CVH mice express at least 1 of the *Tgr5*, *Mrgprc11*, or *Mrgpra3* receptors, compared with only in 62% in healthy states. This suggests alterations in the molecular and functional phenotypes of these neuronal subpopulations in CVH mice, allowing more afferents to be activated by pruritogens compared with healthy states. Correspondingly, we found that using an intracolonic itch cocktail of CCDC, CQ, and BAM8-22 to concurrently activate TGR5, MrgprA3, and MrgprC11 on colonic afferents in CVH mice caused decreases in locomotion and increased grooming and thigmotaxis, indicative of anxiety-like behavior. Interestingly, in addition to altered intestinal motility and chronic pain, IBS patients also suffer from psychiatric conditions, including depression and anxiety (15).

Finally, we show that these TGR5 and MRGPR mechanisms are also present in human DRG neurons. While Mrgprs have been previously detected in human DRG (7, 49), their coexpression profiles with TGR5, TRPA1 and TRPV1 are unclear. Although we could not specifically identify colon-innervating DRG neurons in humans, we could investigate DRG at spinal levels known to innervate the colon (T9-L1), in order to test the concept — both molecularly and functionally — that TGR5-, MrgprX1-, TRPV1-, and TRPA1-coexpressing neurons exist in human DRG. This is important, as CQ induces itch in humans (52), while BAM8-22 produces itch and nociceptive sensations in humans independently of histamine release (53) and TGR5 is linked to cholestatic pruritus in humans (54). As per our findings in mouse DRG, we found that, with single-cell RT-PCR and calcium imaging studies, *TGR5* and *MrgprX1* were expressed in both distinct and overlapping populations of human DRG neurons, which largely coexpressed *TRPV1* and/or *TRPA1*. While there are some discrepancies in absolute percentages between Ca^2+^ imaging and single-cell RT-PCR studies, this could be attributed to translational efficiency of mRNA to protein and surface expression of the receptors at the time of recording. By simulating a pathological state by incubating neurons with inflammatory mediators, significantly increased [Ca^2+^]_i_ responses were observed in human DRG neurons to CCDC, CQ, capsaicin, and AITC compared with normal culture conditions. This suggests, as in our mouse studies, that these neuronal responses to pruritogenic irritants can be readily “tuned” to induce hypersensitive responses in pathological conditions.

Overall, our findings shed new light on the mechanisms contributing to colonic afferent hypersensitivity in healthy and disease-relevant states. We identify mechanisms by which MrgprA3, MrgprC11, and the bile acid receptor TGR5 contribute to the induction of visceral hypersensitivity and altered behavior in response to known pruritogens. Our findings add to the recent discovery of an endogenous mediator, 5-Oxo-eicosatetraenoic acid (5-oxoETE), which activates afferents via a related MRGPR, MRGPRD, to evoke visceral hypersensitivity (55). Our findings demonstrate that the roles of TGR5, MrgprA3, and MRGPRC11 extend beyond itch sensation in the skin, adding to recent work demonstrating that MrgprC11 expressed on vagal sensory neurons contributes to bronchoconstriction and airway hyperresponsiveness (56). Our findings also demonstrate translatability of these TGR5 and Mrgpr mechanisms and their coexpression with TRPV1 and TRPA1 to human DRG neurons. Accordingly, targeting the TGR5- and MRGPR-dependent mechanisms may prove useful in treating visceral hypersensitivity associated with common intestinal disorders.

## Methods

For extensive descriptions of the methodology, please see the Supplemental Material.

### Animals.

Male C57BL/6J mice aged 13–17 weeks were used for studies and acquired from an in-house C57BL/6J breeding programme (strain no. 000664; originally purchased from The Jackson Laboratory, MP14) within SAHMRI’s specific and opportunistic pathogen-free animal care facility. Some experiments also utilized male *Tgr5*^–/–^ (6), *Trpa1*^–/–^ (22), and *Mrgpr-cluster^–/–^* mice (8) or mice over expressing *Tgr5* *(Tgr5-Tg)* (6) from in-house breeding colonies at SAHMRI. *Tgr5^–/–^* and *Tgr5-Tg* mice were gifts originally provided by Johan Auwerx and Kristina Schoonjans, Ecole Polytechnique de Lausanne (Lausanne, Switzerland). *Mrgpr-cluster^–/–^* mice were gifts from Xinzhong Dong (Johns Hopkins University). *Trpa1^–/–^* mice were gifts originally from David Corey (Harvard University, Cambridge, Massachusetts, USA).

### Mouse model of CVH.

Mice were administered intracolonic TNBS and developed colitis (25–27, 36, 38), which healed over 7 days. These mice subsequently developed chronic colonic afferent hypersensitivity (25–27, 36, 38).

### Ex vivo single fiber colonic nociceptor recordings.

Recordings were made from healthy, CVH, or *Tgr5^–/–^*,*Tgr5-Tg*, or *Trpa1^–/–^* mice using standard protocols (25–27, 34, 38). Mechanosensitivity was determined before and after a 5-minute application of OA (100 μM), DCA (100 μM), CCDC (100 μM), BAM8-22 (20 μM), CQ (10 M), or NPFF (5 μM).

### qPCR for pruritogenic receptors in mouse colonic epithelial cells.

The epithelial layer was removed from the colon, and RNA was extracted. qPCR was performed using commercially available hydrolysis TaqMan probes for *Tgr5, Mrgpra3, Mrgprc11,* and *Gucy2c* (GC-C; Supplemental Table 1). Relative abundance was calculated using the ΔCq method (25).

### Retrograde tracing to label the cell bodies of colon-innervating afferents.

Dicarbocyanine dye,1,1-dioctadecyl-3,3,3,3-tetramethlindocarbocyanine methanesulfonate (DiI, 2% in ethanol; Invitrogen) or cholera toxin subunit B conjugated to AlexaFluor-555 (CTB-555; Invitrogen) was injected at 3 sites subserosally within the distal colon. Animals were left to recover for 7–10 days or 4 days, respectively, to identify cell bodies within the DRG (25, 31, 34).

### Single-cell RT-PCR of colon-innervating DRG neurons from healthy and CVH mice.

Individual retrogradely traced colon-innervating DRG neurons (97 from 7 healthy mice and 46 from 4 CVH mice) were picked, RNA was isolated, and mRNA expression was determined in each neuron for *tgr5, MrgprA3, MrgprC11, trpv1,* and *trpa1* using probes indicated within the Supplemental Methods (25).

### [Ca^2+^]_i_ assays of colon-innervating DRG neurons from healthy mice.

Neurons were enzymatically dissociated, plated onto coverslips, and cultured overnight. Neurons were loaded with Fura-2 AM (2 μM), and fluorescence was measured at 340 nm and 380 nm excitation and 530 nm emission (10). Neurons were tested with DCA (100 μM), CCDC (100 μM), TLCA (100 μM), CQ (10 μM), or BAM8-22 (20 μM) and then AITC (100 μM), capsaicin (1 μM), and KCl (50 mM).

### Visualization of pERK neurons within the DH of the spinal cord following CRD.

C57BL/6J healthy (32), CVH (25–27, 30), *Trpa1*^–/–^ (21, 22), *Tgr5*^–/–^ (6, 41), or *Tgr5-Tg* (6, 41) mice were briefly anesthetized with isoflurane anesthetic and a 100-μl enema of either CCDC (100 μM), CQ (10 μM), or saline (vehicle) administered intracolonically via a catheter. Subsequently, a 4-cm balloon catheter was inserted into the perianal canal, and 40 mmHg CRD was performed (10 seconds on, 5 second deflation, repeated 5 times). In separate experiments, an enema of CQ (10 μM) was applied for 5 minutes. After anesthetic overdose, mice were fixed by transcardial perfusion of 4% paraformaldehyde. The spinal cord was then removed and cryoprotected. Frozen sections were cut and incubated with monoclonal-rabbit anti-pERK (4370, Cell Signaling Technology; AB_2315112) and visualized with AlexaFluor-488 (A-21441, Molecular Probes, ThermoFisher Scientific) (25–27, 30–32).

### In vivo VMR to CRD.

Visceral sensitivity to CRD (20, 40, 50, 60, 70, and 80 mmHg, each 20-second durations, applied at 4-minute intervals) was assessed using abdominal electromyography (EMG) in fully awake healthy (30, 33, 34), *Tgr5^–/–^*, *Mrgpr-cluster^–/–^,* or *Trpa1^–/–^* mice, following intracolonic administration (100 μl) of either CCDC (100 μM), CQ (10 μM), BAM8-22 (20 μM), or an intracolonic itch cocktail consisting of a combination of CCDC (100 μM), BAM8-22 (20 μM), and CQ (10 μM). Colonic compliance was assessed by applying graded volumes (40–200 μl, 20-second duration) (33, 34).

### In vivo assessment of animal behavior.

Behavioral testing was evaluated using a behavioral spectrometer (Behavior Sequencer, Behavioral Instruments and BiObserve) (57). Healthy or CVH mice were briefly anesthetized with isoflurane, and a 100-μl enema of an itch cocktail, consisting of CCDC (100 μM), BAM8-22 (20 μM), and CQ (10 μM), was administered intracolonically via a lubricated catheter. A 100-μl saline enema was used as control. Mice were individually placed in the center of the behavioral spectrometer, and their behavior was filmed, tracked and evaluated, and analyzed by a computerized video tracking system (Viewer^3^, BiObserve) for a total of 20 minutes.

### Human tissue.

Human DRG were acquired from 5 organ donors with whole ganglia processed for downstream qPCR or dissociated for single-cell RT-PCR analysis or Ca^2+^ imaging (25, 30). Human colonic biopsies from 15 healthy subjects were acquired from UCLA, recruited primarily by community advertisement.

### mRNA analysis of pruritogenic targets from human tissue.

RNA was extracted from colonic biopsies from 15 subjects and whole bilateral DRG from 4 donors. qPCR was performed using EXPRESS One-Step Superscript qPCR Kit reagents (Invitrogen) with commercially available TaqMan probes for *TGR5*, *MrgprX1*, *Gucy2c*, *TRPA1*, and *TRPV1* (Supplemental Table 1). Relative abundance was estimated using *Δ*Cq method (25).

### Single-cell RT-PCR of human DRG neurons.

A total of 53 human DRG neurons from 4 adult organ donors were individually picked. Ambion Single Cell-to-CT Kit (Invitrogen) was used on an Applied Biosystems 7500 Real-Time PCR System, with the TaqMan primers (Supplemental Table 1) to determine mRNA expression in each neuron for *TGR5*, *MRGPRX1* (human ortholog of mouse *Mrgpra3* and *Mrgprc11*), *TRPV1*, and *TRPA1*.

### [Ca^2+^]_i_ assays of human DRG neurons in response to pruritogens.

Human DRG were dissociated, and neurons were plated on coverslips and cultured. Some coverslips were cultured in normal media, while others — in order to mimic a pathological state — were preincubated with an “inflammatory soup” containing 10 μM each of histamine (MilliporeSigma), PGE II (Tocris), serotonin (Tocris), and bradykinin (MilliporeSigma) 2 hour prior to the experiments at 37°C. For the Ca^2+^ imaging experiments, neurons were loaded with 3 μM Fluo-8 AM, and responses to CCDC (100 μM), CQ (1 μM), BAM8-22 (2 μM), NPFF (2 μM), capsaicin (100 nM), and AITC (50 μM) were determined.

### Statistics.

Data are expressed as mean ± SEM or the percentage of neurons/afferents. Figures were prepared in GraphPad Prism 8 Software. *N* equals the number of animals, while *n* equals the number of neurons/afferents. A *P* value less than 0.05 was considered significant. Differences were indicated significant at levels of *P < 0.05,**P < 0.01,***P < 0.001,****P < 0.0001. VMR to CRD data were statistically analyzed by generalized estimating equations followed by LSD post hoc test using SPSS 23.0 (IMB). All other data were analyzed using GraphPad Prism 8 and analyzed if the data were normally distributed using Kolmogorov-Smirnov or Shapiro-Wilk tests. These data were then analyzed using either (a) 1-way ANOVA, with post hoc analysis conducted by making all possible comparisons among the treatment groups with the Tukey’s tests; (b) 2-way ANOVA, with Bonferroni post hoc analysis conducted by making all possible comparisons among the treatment groups; (c) paired or (d) unpaired 2-tailed *t* tests; or (e) χ^2^ analysis. The specific tests used to analyze each data set is indicated within the individual figure legends.

### Study approval.

All animal experiments were approved and conformed to regulatory standards and the ARRIVE guidelines. The Animal Ethics Committees of the SAHMRI, Flinders University, The University of Adelaide, and Monash University approved all experiments involving animals. All animal experiments conformed to the relevant regulatory standards and the ARRIVE guidelines. All human tissues used for the study were obtained by legal consent from organ donors in the United States. For DRG studies, the DRG were acquired from 5 organ donors with ethical consent. AnaBios Corporation’s procurement network includes only US-based organ procurement organizations and hospitals. Policies for donor screening and consent are the ones established by the United Network for Organ Sharing (UNOS). Organizations supplying human tissues to AnaBios follow the standards and procedures established by the US Centres for Disease Control (CDC) and are inspected biannually by the Department of Health and Human Services (DHHS). Tissue distribution is governed by IRB procedures and compliance with HIPAA regulations regarding patient privacy. All transfers of donor organs to AnaBios are fully traceable and periodically reviewed by US federal authorities. For human colonic biopsies, study approval was obtained from UCLA IRBs (IRB 12-001731), and all subjects signed a written informed consent form prior to starting the study.

## Author contributions

JC, LG, and SMB designed, performed, and analyzed the colonic afferent recordings. AMH, JM, TO, and SMB designed, performed, and analyzed the pERK DH studies. JC, JM, GS, and SMB designed, performed, and analyzed the VMR to CRD studies. GS and SMB designed and performed the behavioral studies. TML, SGC, NWB, and SMB designed, performed, and analyzed the mouse single-cell RT-PCR experiments. TML, DPP, NWB, and SMB designed, performed, and analyzed the mouse Ca^2+^ imaging experiments. SGC and SMB designed, performed, and analyzed the human DRG neuron single-cell PCR and whole human DRG qPCR expression studies. LC collected and provided human colonic biopsies. XD provided *Mrgpr-cluster^–/–^* mice. MSS and XD provided intellectual input on interpretation of the data. ALL and SMB designed, performed, and analyzed the mouse colonic mucosal and the human biopsy qPCR expression studies. PM, AG, and SMB designed, performed, and analyzed the human DRG Ca^2+^ imaging studies. All authors contributed to the discussion and interpretation of the results. SMB wrote the manuscript, with contributions and suggestions from all authors.

## Supplementary Material

Supplemental data

## Version 1 09/19/2019

In-Press Preview

## Version 2 10/17/2019

Electronic publication
