# Supplementary material for: Activation of pruritogenic TGR5, MrgprA3, and MrgprC11 on colon-innervating afferents induces visceral hypersensitivity
Source: JCI Insight. 2019 Oct 17;4(20):e131712. doi: 10.1172/jci.insight.131712 (PMC6824308; doi:10.1172/jci.insight.131712)
Supplement: Supplemental data [file jciinsight-4-131712-s140.pdf]

Supplementary information

Activation of pruritogenic TGR5, MRGPRA3 and MRGPRC11 on colon-innervating afferents induces visceral hypersensitivity

[1,2,3] Joel Castro, [1,2,3] Andrea M. Harrington, [4] TinaMarie Lieu, [1,2,3] Sonia Garcia-Caraballo, [1,2,3] Jessica Maddern, [1,2,3] Gudrun Schober, [1,2,3] Tracey O'Donnell, [1,2,3] Luke Grundy, [1,2,3] Amanda L. Lumsden, [4] Paul Miller, [4] Andre Ghetti, [5,6,7] Martin S. Steinhoff, [8,9,10] Daniel P. Poole, [11] Xinzhong Dong, [12] Lin Chang, [13,14,15] Nigel W. Bunnett, [1,2,3] Stuart M. Brierley

[1] Visceral Pain Research Group, Centre for Neuroscience, College of Medicine and Public Health, Flinders University, Bedford Park, South Australia, 5042, AUSTRALIA. [2] Hopwood Centre for Neurobiology, Lifelong Health Theme, South Australian Health and Medical Research Institute (SAHMRI), North Terrace, Adelaide, South Australia 5000, AUSTRALIA, [3] Centre for Nutrition and Gastrointestinal Diseases, Discipline of Medicine, University of Adelaide, North Terrace, Adelaide, South Australia 5000, AUSTRALIA. [4] AnaBios Corporation, San Diego, CA, USA. [5] Department of Dermatology and Dermatology Immunology Institute, Hamad Medical Corporation, Doha, Qatar; [6] Department of Dermatology, Weill Cornell Medicine-Qatar and Weill Cornell University New York, USA; [7] School of Medicine Qatar University, Doha, Qatar. [8] Monash Institute of Pharmaceutical Sciences, and [9] Australian Research Council Centre of Excellence in Convergent Bio-Nano Science and Technology, Monash University, Parkville, VIC 3052, AUSTRALIA. [10] Department of Anatomy and Neuroscience, University of Melbourne, Parkville, VIC 3010, AUSTRALIA. [11] The Solomon H. Snyder Department of Neuroscience, Center for Sensory Biology, School of Medicine, Howard Hughes Medical Institute, Johns Hopkins University, Baltimore, Maryland, USA. [12] G. Oppenheimer Centre for Neurobiology of Stress and Resilience, David Geffen School of Medicine at UCLA, UCLA, 10833 Le Conte Avenue, CHS 42-210, Los Angeles, CA 90095-7378, USA. [13] Department of Pharmacology and Therapeutics, University of Melbourne, Parkville, VIC 3010, AUSTRALIA. [14] Departments of Surgery and [15] Pharmacology Columbia University, New York, NY 10032, USA.

Corresponding Author: Prof. Stuart M. Brierley, Ph.D. Visceral Pain Research Group, Level 7, South Australian Health and Medical Research Institute (SAHMRI), North Terrace, Adelaide, SA 5000, AUSTRALIA. Email: stuart.brierley@flinders.edu.au Phone: +61 8 8128 4848

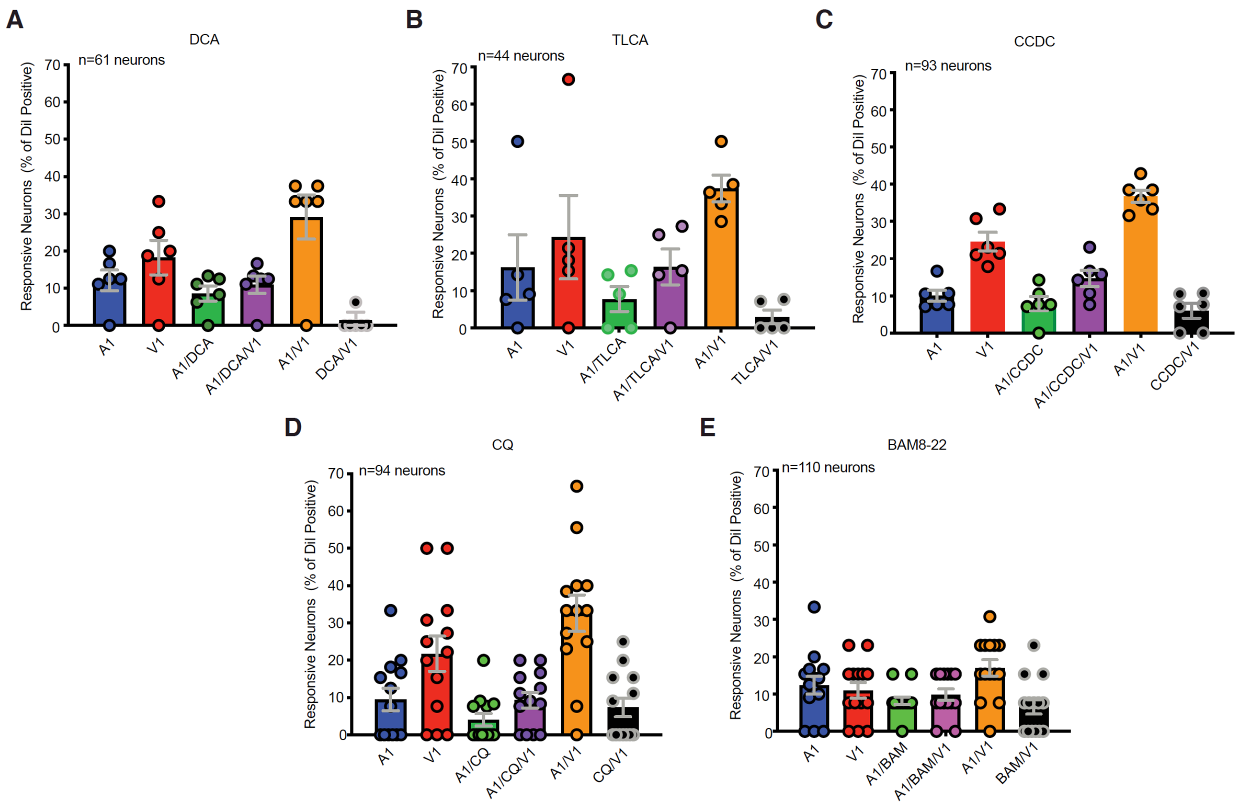

<u>Supplementary Figure 1:</u> Functional co-expression of pruritogenic G protein-coupled receptors (GPCRs) and TRP channels using Ca^{2+} imaging in mouse colon-innervating DRG neurons.

All data represent the percentage of DiI positive (colon-innervating) DRG neurons from individual coverslips from 6 mice responding to: **A)** The TGR5 agonist deoxycholic acid (DCA): Data are calculated from 61 colon-innervating DRG neurons tested with AITC (A1: 100μM), DCA (100μM), and capsaicin (V1: 1μM) in sequential order. A1/DCA represents neurons responding to both AITC and DCA. A1/DCA/V1 represents neurons responding to AITC, DCA and capsaicin. A1/V1 represents neurons responding to AITC and capsaicin and DCA/V1 neurons responding to DCA and capsaicin. **B)** The TGR5 agonist taurolithocholic acid (TLCA): Data are calculated from 44 colon-innervating DRG neurons tested with AITC (A1: 100μM), TLCA (100μM), and capsaicin (V1: 1μM) in sequential order. **C)** The TGR5 agonist CCDC: Data are calculated from 93 colon-innervating DRG neurons tested with AITC (100μM), CCDC (100μM), and capsaicin (1μM) in sequential order. **D)** The MRGPRA3 agonist CQ: Data are calculated from 94 colon-innervating DRG neurons tested with AITC (A1: 100μM), CQ (10μM), and capsaicin (V1: 1μM) in sequential order. **E)** The MRGPRC11 agonist BAM8-22: Data are calculated from 110 colon-innervating DRG neurons tested with AITC (A1: 100 μM), BAM8-22 (BAM: 2μM), and capsaicin (V1: 1μM) in sequential order. Data represent Mean ± SEM. All data are from N=5-7 mice.

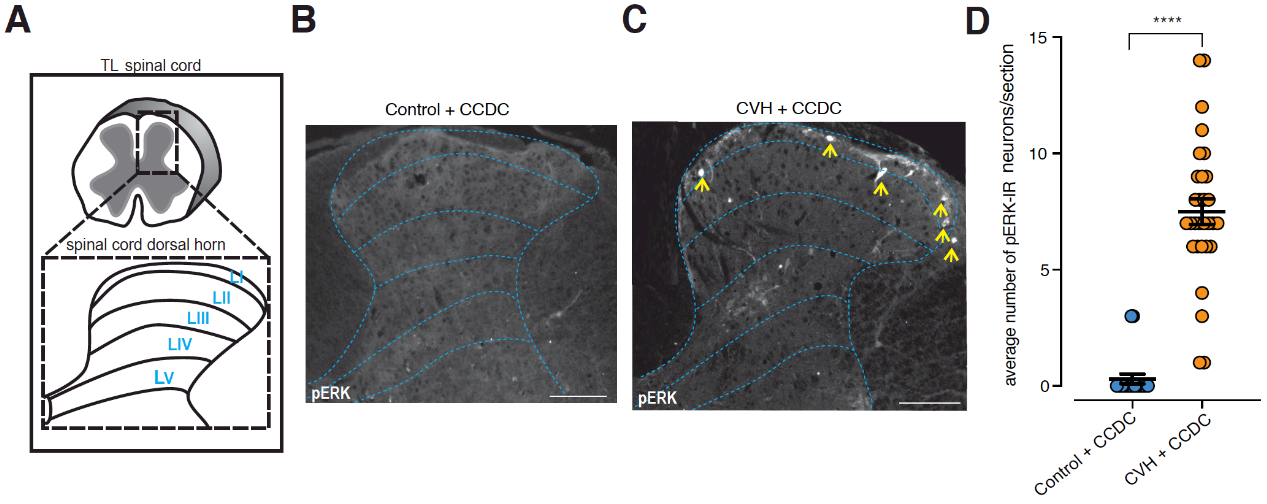

<u>Supplementary Figure 2:</u> In vivo intra-colonic administration of CCDC induces activation of dorsal horn neurons within the spinal cord of CVH mice.

A) Schematic representation of laminae I–V (LI–LV) in the dorsal horn of the thoracolumbar spinal cord. **B)** Lack of pERK-immunoreactive (pERK-IR) neurons in the dorsal horn of healthy control mice administered an intra-colonic enema of CCDC (100μl bolus of 100μM). **C)** In contrast, CVH mice display numerous pERK-IR activated dorsal horn neurons in response to intra-colonic CCDC administration (100μM). Yellow arrows indicate pERK-IR neurons. **D)** Grouped data showing the average number of pERK-IR neurons in the dorsal horn of healthy control and CVH mice in response to intra-colonic CCDC (100μM) administration (****P<0.0001, dots indicate individual counts in spinal cord sections from Control mice + CCDC: N=4, and CVH mice + CCDC: N=4. Data represent Mean ± SEM. *P* values are based on an un-paired t-test. Scale bars in **B,C** equal 100 μm.

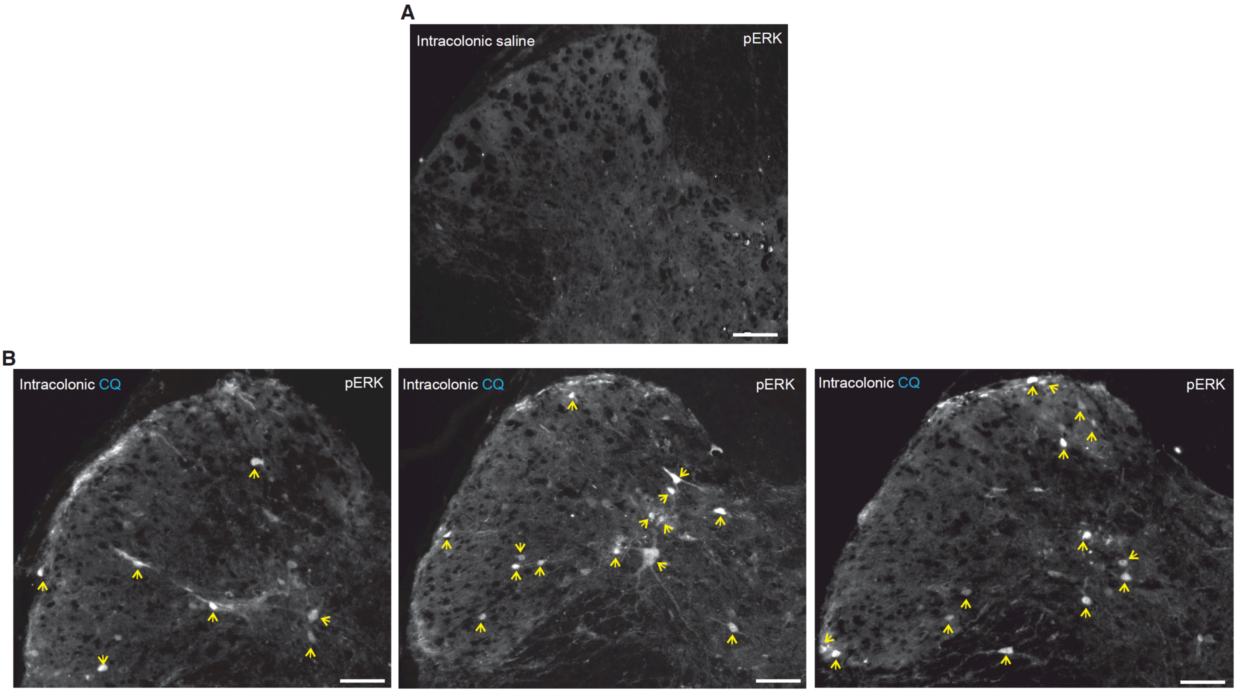

<u>Supplementary Figure 3:</u> In vivo intra-colonic administration of chloroquine (CQ) induces activation of dorsal horn neurons within the spinal cord of CVH mice.

A) Intra-colonic administration of saline (100μl) does not result in pERK-immunoreactivity (pERK-IR) within the dorsal horn of the thoracolumbar spinal cord. **B)** In contrast intracolonic administration of chloroquine (CQ; 100μl bolus of 10μM) causes pronounced activation of dorsal horn neurons, as indicated by pERK-IR (yellow arrows). This is particularly apparent within laminae I, III, IV and V. Scale bars in equal 50 μm.

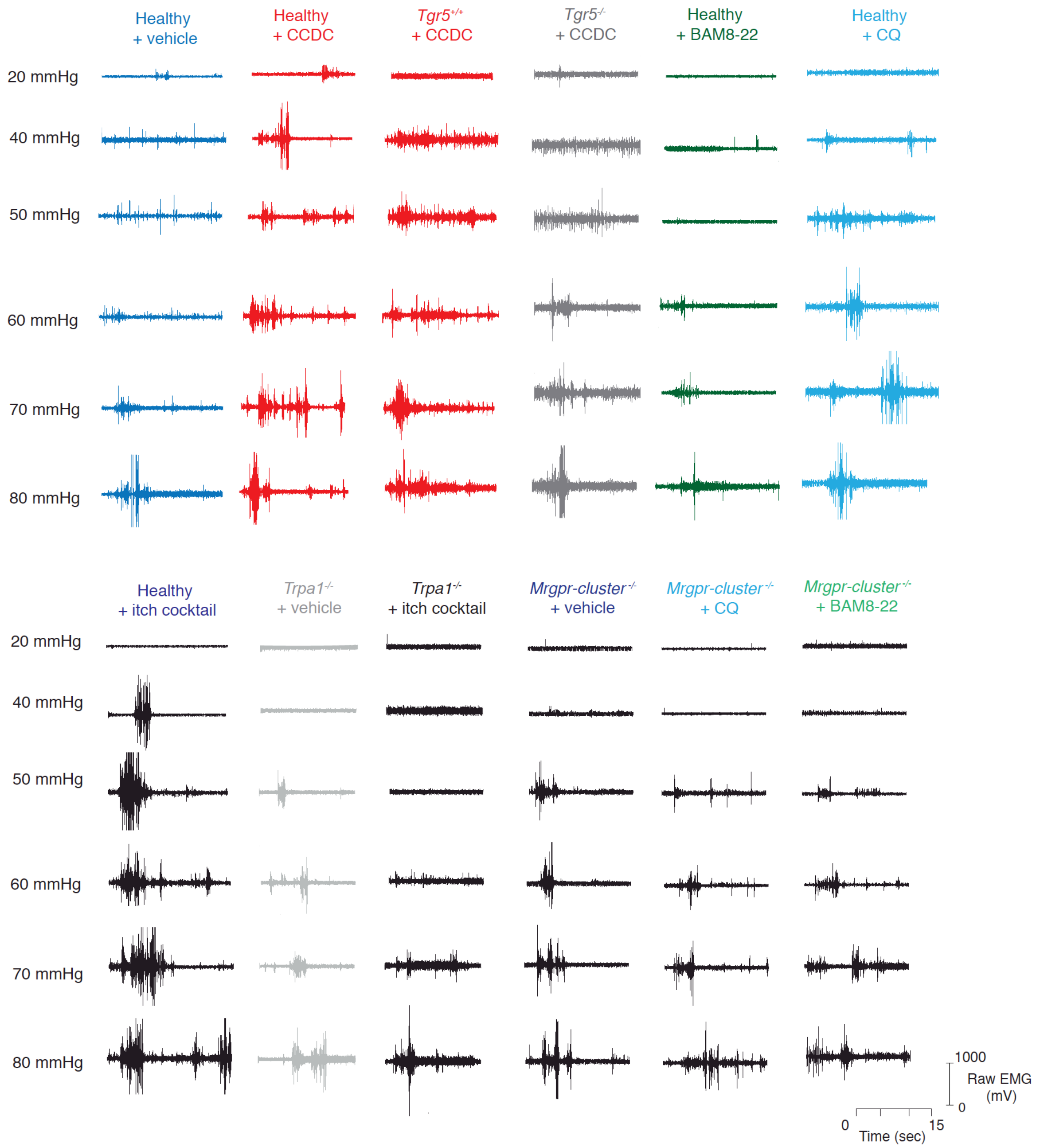

<u>Supplementary Figure 4:</u> Representative tracings of the electromyographically (EMG) recorded VMR to 20, 40, 50, 60, 70 and 80 mmHg of CRD in healthy control, *Tgr5*$^{-/-}$, *Trpa1*$^{-/-}$ and *Mrgpr cluster* $^{-/-}$ mice pretreated in vivo with combinations of intra-colonically administered vehicle (saline), CCDC (100µM), BAM8-22 (20µM), CQ (10µM), or an 'itch cocktail' consisting of a combination of CCDC (100µM), BAM8-22 (20µM) and CQ (10µM). For analysis, the raw EMG signal was integrated and the VMR was expressed as the AUC of the EMG signal during distension, corrected for the AUC during baseline.

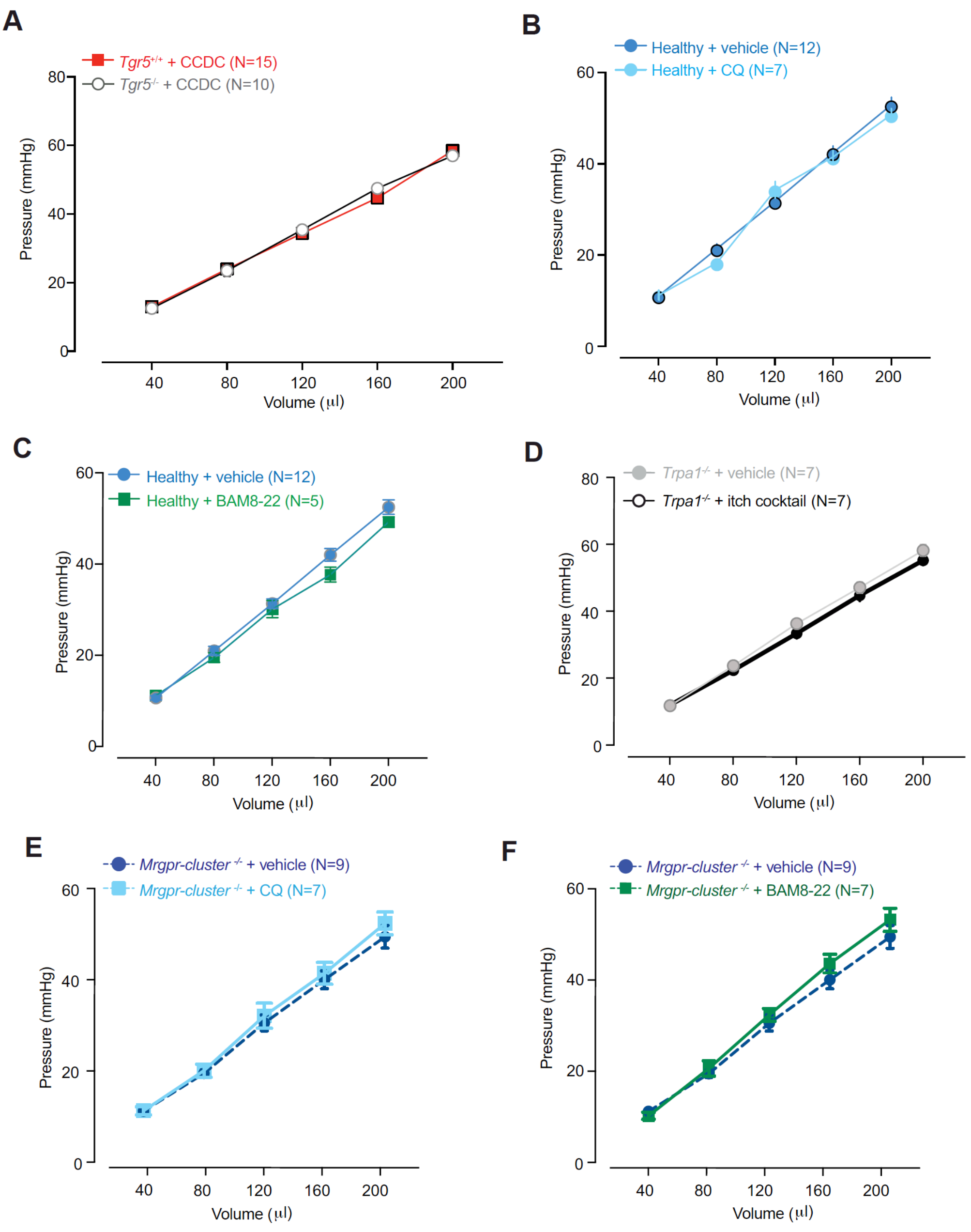

Supplementary Figure 5: Colonic compliance is unaltered by pruritogenic agonists.

Colonic compliance was not affected by intra-colonic administration of **A)** 100µM CCDC (*Tgr5*$^{+/+}$ + CCDC: N=15; *Tgr5*$^{-/-}$ + CCDC: N=10), **B)** 10µM CQ (Healthy + vehicle: N=12; Healthy + CQ: N=7), **C)** 20µM BAM8-22 (Healthy + vehicle: N=12; Healthy +BAM8-22: N=5) or by **D)** an itch cocktail consisting of a combination of CCDC, BAM8-22 and CQ (*Trpa1*$^{-/-}$ + vehicle: N=7; *Trpa1*$^{-/-}$ + itch cocktail: N=7). **E-F)** Colonic compliance was also unaltered in *Mrgpr cluster*$^{-/-}$ mice intra-colonically administered with **E)** 10µM CQ (*Mrgpr cluster*$^{-/-}$ + vehicle: N=5 vs. *Mrgpr cluster*$^{-/-}$ + CQ: N=5) or **F)** 20µM BAM8-22 (*Mrgpr cluster* $^{-/-}$ + vehicle: N=5 vs. *Mrgpr cluster*$^{-/-}$ + BAM8-22: N=5). This suggested the effects induced by the intra-colonic applied agonists are not mediated by changes in muscle contractility and that they occur at the level of the sensory afferent. Data are presented as Mean ± SEM. *P* values are based on two-way ANOVAs.

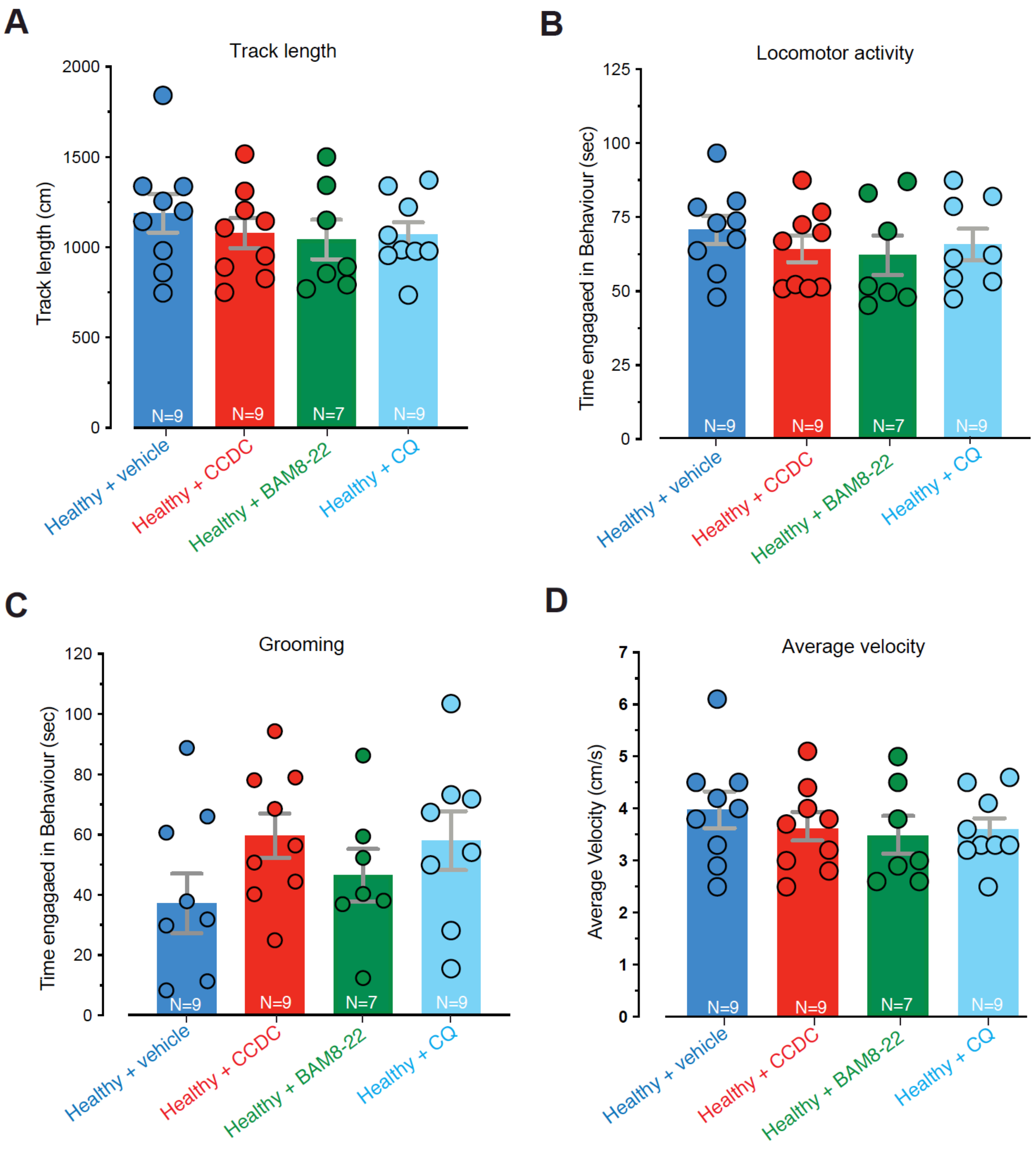

<u>Supplementary Figure 6</u>: Intra-colonic administration of the individual pruritogenic agonists of TGR5, MRGPRC11 and MRGPRA3 does not induce significant changes in animal behaviour in healthy control mice.

Group data from behavioural studies showing that the **A)** track length covered, **B)** locomotor activity, **C)** grooming and **D)** average velocity was not significantly altered in mice administered intra-colonic CCDC (100µM; N=9), BAM8-22 (20µM; N=7), or CQ (10µM; N=9) compared with intra-colonic administration of vehicle (saline; N=9). Data presented are Mean ± SEM. Each dot represents data from an individual animal. Analysis was performed by one-way ANOVA.

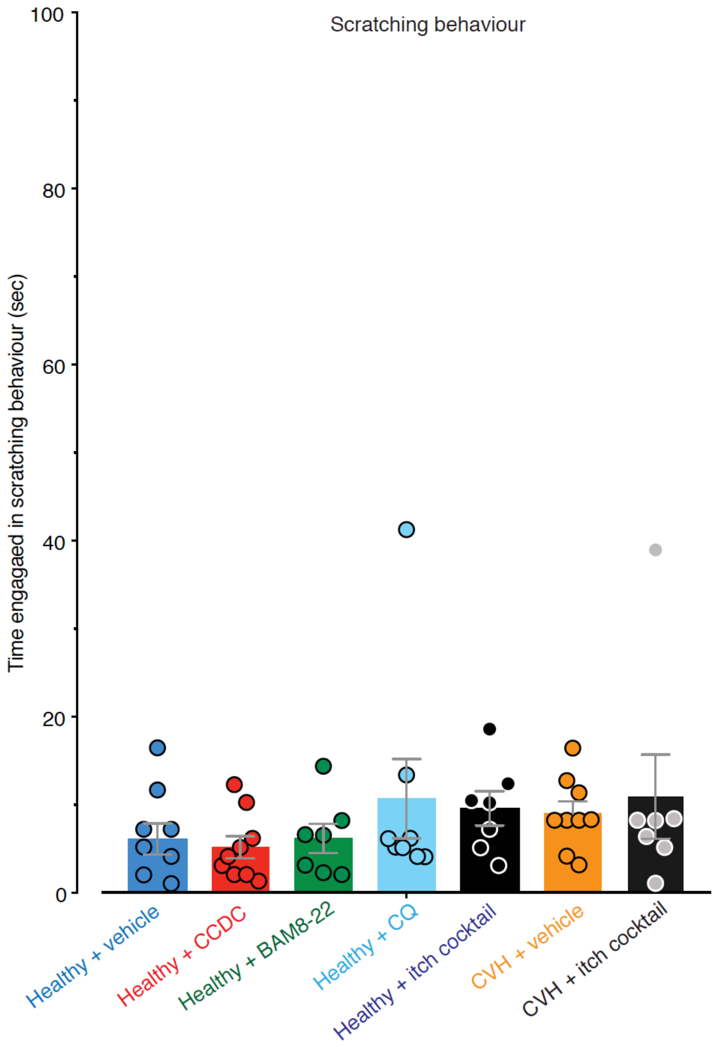

<u>Supplementary Figure 7</u>: Intra-colonic administration of pruritogenic agonists does not induce scratching behaviour in healthy control nor CVH mice.

Group data from behavioural studies showing that the number of scratching events was not altered in mice administered intra-colonic CCDC (100μM; N=9), BAM8-22 (20μM; N=7), CQ (10μM; N=8), or an itch cocktail of CCDC, BAM8-22 and CQ (Healthy: N=7; CVH: N=7) compared with intra-colonic administration of vehicle (saline; Healthy: N=8; CVH: N=9). Data presented are Mean ± SEM. Analysis was performed by one-way ANOVA.

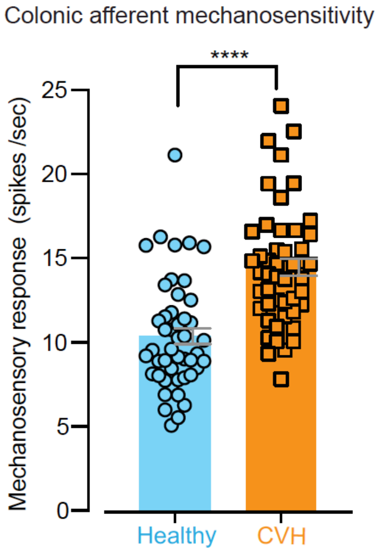

<u>Supplementary Figure 8</u>: Colonic afferents from CVH mice exhibit pronounced mechanical hypersensitivity.

Combined group data from 47 healthy colonic afferents (combined data from Figure 1A, 1B, 1C, 1G, 1H, 1I) and 49 CVH colonic afferents (combined data from Figure 7A, 7B, 7C, 7D, 7E, 7F). Un-paired two tailed t-tests indicate that CVH afferents display significantly increased action potential firing to mechanical stimulation compared with healthy afferents. Data presented are Mean ± SEM.

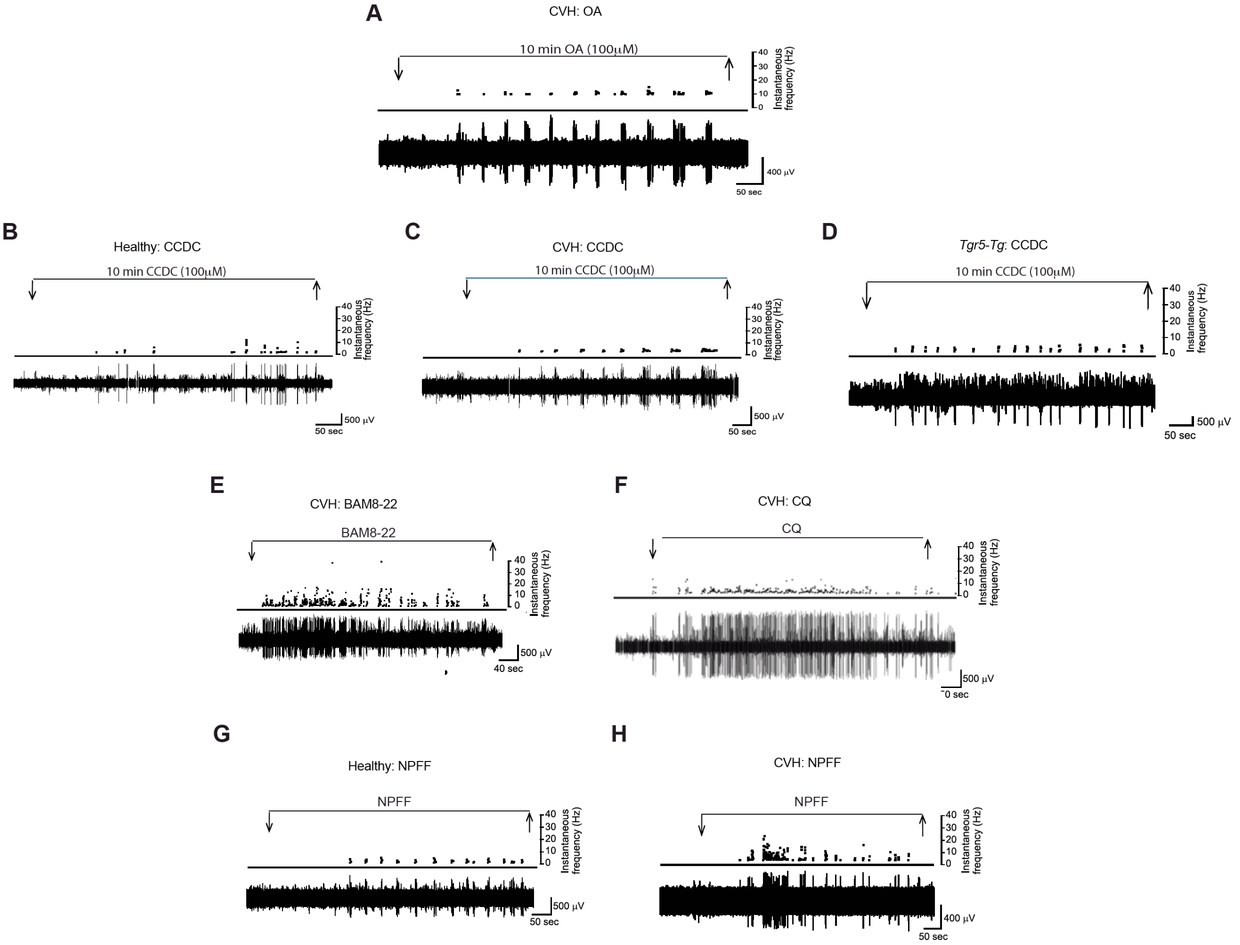

<u>Supplementary Figure 9</u>: Application of TGR5 agonists and MRGPR agonists activates colonic afferents.

Representative examples showing that **A)** oleanoic acid (OA; 100μM) induces action potential firing in a subset of nociceptors from CVH mice. This was not observed with OA application to nociceptors from control mice (not shown). **B)** CCDC (100μM) application evoked responses in a small subset of colonic nociceptors from healthy mice with enhanced responses to CCDC in nociceptors from **C)** CVH and **D)** TGR5 over-expressing (*Tgr5-Tg*) mice. **E)** Application of the MRGPRC11 agonist BAM8-22 (20μM) evoked action potential firing in a subset of nociceptors from CVH, but not control mice (not shown). **F)** Similarly, application of the MRGPRA3 agonist chloroquine (CQ; 10μM) induced action potential firing in a subset of colonic nociceptors from CVH mice. **G)** The MRGPRC11/A4 agonist NPFF (5μM) evoked responses in a subset of nociceptors from healthy mice with **H)** enhanced responses to NPFF in nociceptors from CVH mice.

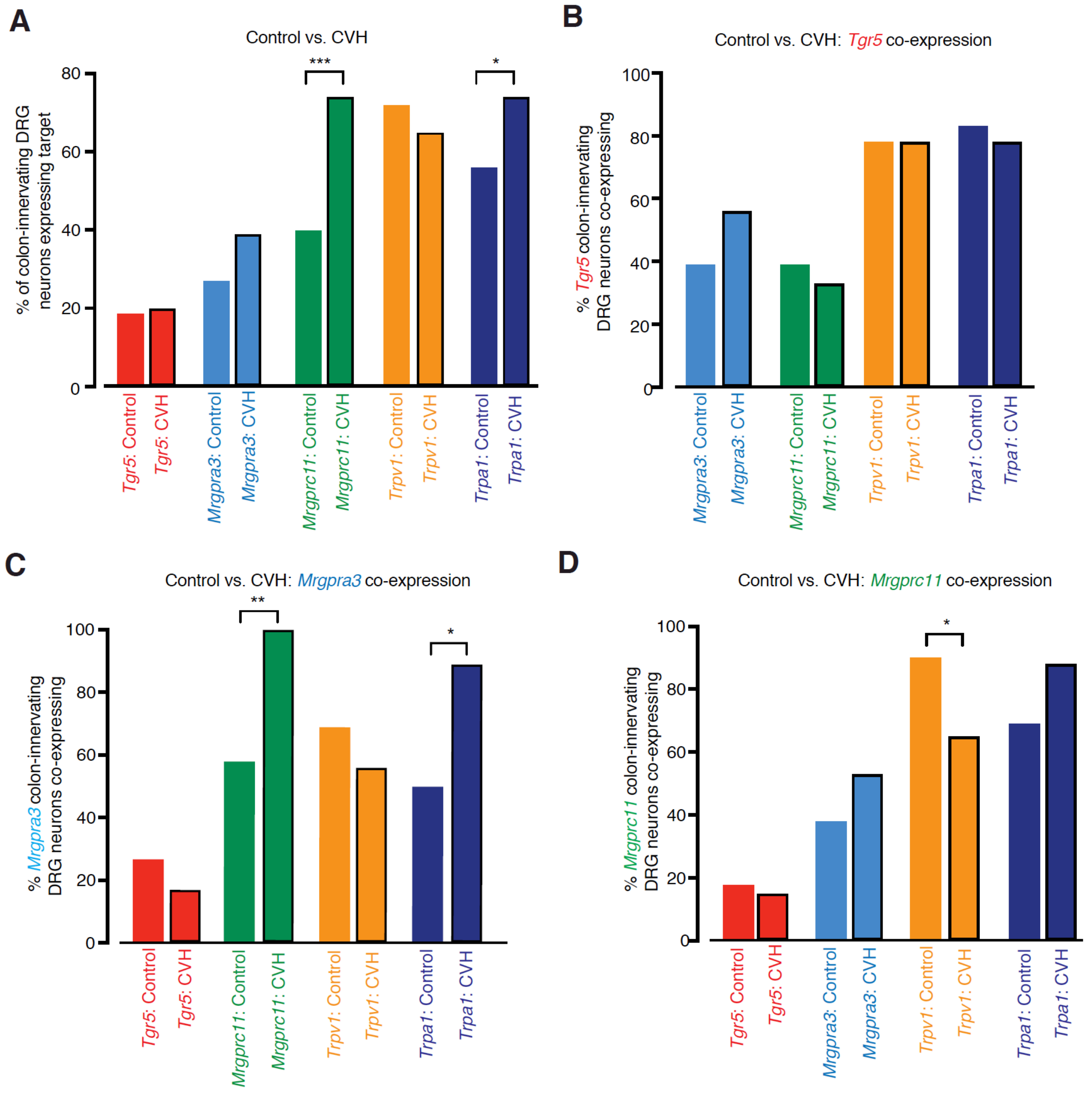

<u>Supplementary Figure 10:</u> Comparison of *Tgr5*, *Mrgpra3, Mrgprc11*, *Trpv1* and *Trpa1* expression and co-expression in colon-innervating DRG neurons from healthy and CVH mice.

A) Significantly more colon-innervating DRG neurons from CVH mice display expression for both *Mrgprc11* (****P*<0.001) and *Trpa1* (**P*<0.05) compared with healthy control mice. **B)** *Tgr5* co-expression with *Mrgpra3, Mrgprc11*, *Trpv1* and *Trpa1* was unaltered in colon-innervating DRG neurons from CVH mice compared with healthy control mice. **C)** In CVH mice significantly more *Mrgpra3* expressing colon-innervating DRG neurons co-express *Mrgprc11* (***P*<0.01) and TRPA1 (**P*<0.05) compared with healthy control mice. **D)** Significantly fewer *Mrgprc11* expressing colon-innervating DRG neurons co-express *Trpv1* in CVH mice (**P*<0.05). All data were analysed, and significance determined by χ^2 tests. Percentages are calculated based on the data presented in Figure 2C and Figure 8B.

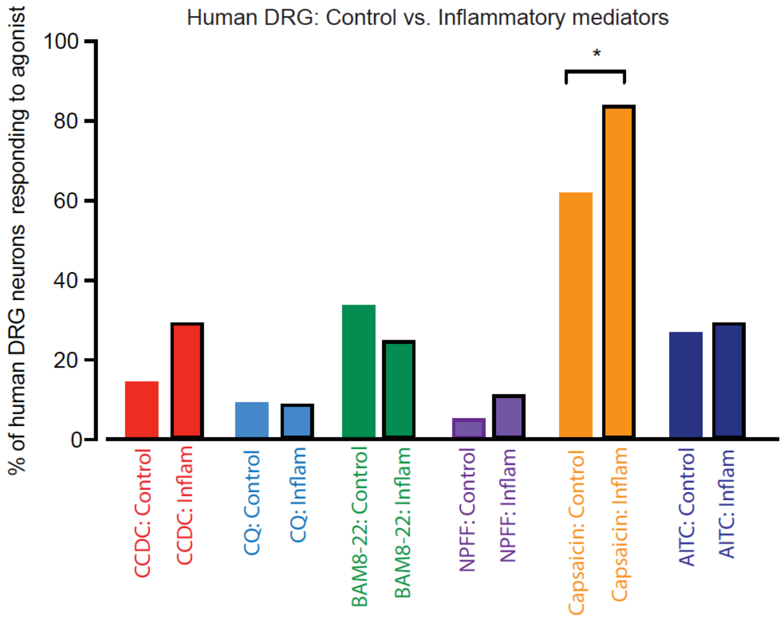

Supplementary Figure 11: Comparison of human DRG neuron responsiveness to agonists following incubation with control media or inflammatory mediators. Significantly more human DRG neurons incubated with inflammatory mediators responded to capsaicin (*$P<0.05$) compared with neurons incubated with control media. All data were analysed, and significance determined by χ^2 tests. Percentages are calculated based on the data presented in Figure 11H, and Figure 11I.

Target	Gene	Aliases	Product code	Product size	NCBI accession #
Mas-related GPR, member X1	*MRGPRX1*	GPCR	Hs00600918_s1	93	NM_147199.3
G protein-coupled bile acid receptor 1	*GPBAR1*	TGR5	Hs01937849_s1	159	NM_001077191.1
tubulin, beta 3 class III	*Tubb3*		Hs00964962_g1	56	NM_001197181.1; NM_006086.3
glial fibrillary acidic protein	*GFAP*		Hs00909233_m1	57	NM_001131019.2; NM_001242376.1; NM_002055.4
transient receptor potential cation channel, subfamily V, member 1	*TRPV1*	VR1	Hs00218912_m1	94	NM_018727.5; NM_080704.3; NM_080705.3; NM_080706.3
transient receptor potential cation channel, subfamily A, member 1	*TRPA1*	ANKTM1	Hs00175798_m1	124	NM_007332.2
guanylate cyclase 2C	*GUCY2C*	GC-C	Hs00192035_m1	106	NM_004963.3
actin, beta	*ACTB*		Hs01060665_g1	63	NM_001101.3
PPIA (cyclophilin A)	*PPIA*		Hs99999904_m1	98	NM_021130.3
glyceraldehyde-3-phosphate dehydrogenase	*GAPDH*		Hs99999905_m1	122	NM_002046.4

Supplementary Table 1: Human TaqMan® Primers used for mRNA analysis in qRT-PCR studies in 1) colonic mucosal biopsies and 2) whole human DRG and 3) single cell-PCR studies in individual DRG neurons.

SUPPLEMENTARY ONLINE METHODS:

Animals

The Animal Ethics Committees of the South Australian Health and Medical Research Institute (SAHMRI), Flinders University, The University of Adelaide and Monash University approved all experiments involving animals. All experiments conformed to the relevant regulatory standards and the ARRIVE guidelines. For experiments conducted in Adelaide, male C57BL/6J mice at 13-17 weeks of age were used and acquired from an in-house C57BL/6J breeding programme (Jax strain #000664; originally purchased from The Jackson Laboratory (breeding barn MP14; Bar Harbor, ME; USA) within SAHMRI's specific and opportunistic pathogen-free animal care facility. Studies also utilized *Trpa1* null mutant *Trpa1*$^{-/-}$ (1,2), *Tgr5*$^{-/-}$ (3,4), *Mrgpr cluster*$^{-/-}$ (5) and mice over-expressing TGR5 (*Tgr5-Tg*) (3,4), which were also bred within the SAHMRI animal care facility. Mice were group housed (maximum five mice per cage) within individual ventilated cages (IVC), which were filled with coarse chip dust-free aspen bedding (PURA®; Cat# – ASPJMAEB-CA, Niederglatt, Switzerland). These cages were stored on IVC racks in specific housing rooms within a temperature-controlled environment of 22°C and a 12 h light/12 h dark cycle. Mice had free access to LabDiet® JL Rat and Mouse/Auto6F chow (Cat# 5K52, St. Louis, MO; USA) and autoclaved reverse osmosis purified water. For experiments conducted at Monash, male C57BL/6 mice were obtained from the Monash Animal Research Platform and used at 6-12 weeks of age. Mice were maintained in a temperature-controlled environment with a 12 h light/dark cycle and free access to food and water. Mice were killed by anaesthetic overdose and bilateral thoracotomy.

Mouse model of chronic visceral hypersensitivity (CVH)

Colitis was induced by administration of 2,4,6-trinitrobenzenesulfonic acid (TNBS) as described previously (6-10). Briefly, 13-week-old anaesthetized mice were administered a once off intra-colonic enema of 0.1 ml TNBS (130 µL/ml^{-1} in 35% ethanol) via a polyethylene catheter (7,8,11). Previous histological examination of mucosal architecture, cellular infiltrate, crypt abscesses, and goblet cell depletion confirmed significant TNBS-induced damage by day 3 post-treatment, which largely recovered by day 7, and fully recovered by 28 days (11,12). High-threshold colonic nociceptors from mice at the 28-day time point display significant mechanical hypersensitivity, and lower mechanical activation thresholds. Mice from this model also display increased neuronal activation in the dorsal horn of the spinal cord in response to noxious colorectal distension, as well as sprouting of colonic afferent terminals within the dorsal horn (13). This model also induces hyperalgesia and allodynia to colorectal distension (14), and is therefore termed 'Chronic Visceral Hypersensitivity' (CVH) (7-11,15).

Ex vivo single fibre colonic nociceptor recordings and responses to pruritogens

Tissues for ex vivo colonic nociceptor recordings were from healthy or CVH (6-12,16) C57BL/6J mice or *Trpa1*$^{-/-}$ (1,2), *Tgr5*$^{-/-}$ (3,4), *Mrgpr cluster* $^{-/-}$ (5) or *Tgr5-Tg* (3,4) mice. On the day of experimentation, mice were humanely killed by CO_2 inhalation and the colon/rectum (5-6 cm) and attached splanchnic nerves were removed and afferent recordings from splanchnic nerves were performed as described previously (11,17,18). Briefly, colons were removed, dissected open and pinned flat, mucosal side up, in a specialized organ bath. The colonic compartment was superfused with a modified Krebs solution (in mM: 117.9 NaCl, 4.7 KCl, 25 $NaHCO_3$, 1.3 NaH_2PO_4, 1.2 $MgSO_4$ $(H_2O)_7$, 2.5 $CaCl_2$, 11.1 D-glucose), and bubbled with carbogen (95% O_2, 5% CO_2) at a temperature of 34°C. All solutions contained the L-type calcium channel antagonist nifedipine (1μM) to suppress smooth muscle activity and the prostaglandin synthesis inhibitor indomethacin (3μM) to block endogenous prostaglandin production. The nerve bundle was extended into a paraffin-filled recording compartment in which finely dissected strands were laid onto a mirror, and single fibres placed on the platinum recording electrode. Action potentials, generated by mechanical stimuli to the receptive field, were recorded by a differential amplifier, filtered and sampled (20 kHz) using a 1401 interface (Cambridge Electronic Design, Cambridge, UK) and stored on a PC for off-line analysis. Recordings were made using standard protocols (8,9,17). Receptive fields were identified by systematically stroking the mucosal surface of the colon with a stiff brush to activate all subtypes of mechanoreceptors. Categorization of afferent properties was in accordance with our previously published classification system (2,6-11,17,19). Once identified, receptive fields were tested with three distinct mechanical stimuli to enable classification: static probing with calibrated von Frey hairs (vfh) (2 g force; applied 3 times for a period of 3 seconds), mucosal stroking with calibrated vfh (10 mg force; applied 10 times) or circular stretch (5 g; applied for a period of 1 min). We recorded from serosal afferents, also termed vascular afferents (20), from the splanchnic pathway. These colonic afferents have high-mechanical activation thresholds and respond to noxious distension (40 mmHg), stretch (≥7 g) or vfh filaments (2 g) but not to fine mucosal stroking (10 mg vfh) (9,10,17,21). They express and respond to agonists of algesic channels and receptors including TRPV1 (18), TRPA1 (2), TRPV4 (22), PAR_2 (23), $P2X_3$ (18), B1 (24), $Na_V1.1$ (10,25), $Na_V1.8$ (26), and TNFR1 (21). They are therefore referred to as colonic 'nociceptors'. Baseline mechanosensitivity was determined in response to application of a 2g von frey hair (vfh) probe to the afferent receptive field for 3 seconds. This process was repeated 3–4 times, separated each time by 10 seconds. The compounds, oleanolic acid (OA; 100 μM), Deoxycholic acid (DCA; 100μM), 3-(2-chlorophenyl)-N-(4-chlorophenyl)-N,5-dimethyl-4-isoxazolecarboxamide (CCDC; 100μM), BAM8-22 (20 μM), chloroquine (CQ; 10μM), and neuropeptide FF (NPFF; 5μM) were prepared from stock solutions (1% DMSO), and diluted to appropriate final concentrations in Krebs solution. A small chamber was then applied to the mucosal surface of the colon, surrounding the afferent receptive field. Residual Krebs solution within the chamber was aspirated and the respective compounds applied in separate experimental preparations for 5 minutes. Action potential firing was noted and analysed during this time. Mechanical sensitivity was then re-tested in response to application of 3x 3 sec 2g vfh probes to the afferent receptive field. Action

potentials were analyzed off-line using the Spike 2 (version 5.21) software (Cambridge Electronic Design, Cambridge, UK) and discriminated as single units based on distinguishable waveforms, amplitudes and durations. Data are expressed as Mean ± SEM. n = the number of afferents recorded. N = the number of animals used for those specific experiments. Data were statistically compared using Prism 8 software (GraphPad Software, San Diego, CA, USA), and analysed using paired t-tests. CCDC was purchased from Cayman Chemicals (USA), with DCA, OA, BAM8-22, and NPFF purchased from Tocris (UK). Chloroquine, AITC, and capsaicin were purchased from Sigma-Aldrich (Australia).

qRT-PCR for pruritogenic targets within colonic epithelial cells

Colons were excised from mice, cut longitudinally, pinned epithelial side up on a sylgard-coated petri dish and washed 3x in fresh MEM media containing 1% PSF/0.7% HEPES. Colons were incubated with 2.5mg/ml dispase in MEM (containing PSF and HEPES) for 2hrs at room temperature. The epithelial layers were gently scraped with a blunt scalpel and cells immediately placed into 0.25% trypsin-EDTA solution and then incubated at 37°C for 10 minutes. The cell suspension was then centrifuged at 1500rpm for 5 minutes at 4°C, the media aspirated, and the pellet was resuspended in keratinocyte serum-free growth media (K-SFM) media containing 1% PSF (27). RNA was isolated using the PureLink RNA Micro kit (#12183-016, Invitrogen, ThermoFisher Scientific) according to manufacturer's instructions. All samples were DNAse treated (# 12185-010, Life Technologies, ThermoFisher Scientific). A NanoDrop spectrometer determined Quantity and purity. qRT-PCR was performed using EXPRESS One-Step Superscript® qRT-PCR Kit (#11781-01K, Life Technologies, ThermoFisher Scientific) and DEPC-treated water (#AM9916, AMBION, ThermoFisher Scientific). Primers for *Tgr5 (Mm04212121_s1), Mrgpra3 (Mm02620679_s1), Mrgprc11 (Mm02525847_g1), Guc2yc (Mm01267708_m1), Actb (Mm00607939_s1) and Ppia (Mm02342430_g1)*. Samples were run in duplicates. qRT-PCR curves were analysed using 7500 Software v.2.06 from Life Technologies. Quantification cycles (Cq) were exported to Microsoft Excel® and relative abundance to endogenous control genes estimated using delta Cq method, as described previously (2,22,28).

Retrograde tracing to identify DRG neurons innervating the colon

Mice (C57BL/6) were anesthetized with 5% isoflurane. The descending colon was exposed through a midline incision and the overlying viscera were kept moist with saline-soaked gauze. Dicarbocyanine dye,1,1-dioctadecyl-3,3,3,3-tetramethlindocarbocyanine methanesulfonate (DiI, 2% in ethanol; Invitrogen, Carlsbad, CA) was injected into in the wall of the colon (3-4 sites, 2 µL per site) using a Hamilton microliter syringe and a 32-gauge needle. To prevent leakage to other organs, the needle was held in place for 30 seconds after the injection, and any excess dye was carefully removed.

The abdominal wall and skin were closed, and animals were allowed to recover. After 7-10 days, animals were killed and DRG (T10–L1) were removed. Alternatively, cholera toxin subunit B conjugated to AlexaFluor 488 (CTB-488); Invitrogen, Carlsbad, CA) was injected at three sites sub-serosally within the wall of the distal colon of healthy control or CVH mice mice instead of DiI (2,7,9,13). After 4 days, animals were humanely killed by CO_2 inhalation for subsequent thoracolumbar (TL: T10-L1) DRG removal and dissociation.

Single cell RT-PCR of pruritogenic targets within colon-innervating DRG neurons from healthy control and CVH mice

After retrograde tracing, DRG were collected from thoracolumbar regions from healthy and CVH mice and were dissociated as previously described (7,29). Dissociated neurons were plated onto poly D-lysine- (0.1 $mg.ml^{-1}$) and laminin- (0.004 $mg.ml^{-1}$) coated cover slips and were incubated in Leibovitz's L-15 medium containing 10% FBS in a humidified incubator (8 h, 37°C). Individual DiI-positive small diameter (<25 µm) neurons were selected and drawn into a glass-pipette (tip diameter 25-100 µm) by applying negative pressure. The pipette tip was broken in a PCR tube containing 1 µl of resuspension buffer and RNAse inhibitor (RNAseOUT, 2 $U.\mu l^{-1}$, Invitrogen) and snap frozen. PCR reactions used the following intron-spanning mouse primers: *Tgr5* outer primer forward 5'-cactgcccttctctctgtcc-3', reverse 5'-tcaagtccaggtcaaatcctg-3'; *Tgr5* inner primer forward 5'-tgctcttcttgctgtgttgg-3', reverse 5'-gtccctcttggctcttcctc-3' (236bp); *Mrgpra3* forward 5'-cgacaatgacacccacaacaa-3', reverse 5'-ggaagccaaggagccagaac-3', *Mrgprc11* forward 5'-gcctcttgggctttacttgtt-3', reverse 5'-gggacctatgctttctatgctg-3', *Trpv1* forward 5'-tcaccgtcagctctgttgtc-3', reverse 5'-gggtctttgaactcgctgtc-3'; *Trpa1* forward 5'-ggagcagacatcaacagcac-3', reverse 5'-gcaggggcgacttcttatc-3'; or *Actb* forward 5'-ctggtcgtcgacaacggctcc-3', 5'-reverse gccagatcttctccatg-3' (29). As a negative control, superfusing fluid from the vicinity of the collected cells was amplified, or RT was omitted. Products were separated by electrophoresis (2% agarose), stained using ethidium bromide, and sequenced to confirm identity. Differences between the percentage of colon-innervating DRG neurons expressing *Tgr5, Mrgpra3, Mrgprc11, Trpv1, Trpa1* transcripts and their co-expression in healthy and CVH states were analysed, and significance determined by χ^2 tests.

$[Ca^{2+}]_i$ assays of pruritogenic agonist responses within colon-innervating DRG neurons from healthy control mice

Neurons were loaded with Fura-2-AM (2 µM) in assay buffer (mM: NaCl 150, KCl 2.6, $CaCl_2$ 0.1, $MgCl_2$ 1.18, D-glucose 10, HEPES 10, pH 7.4) containing 4 mM probenecid and 0.5% BSA for 30 min at 37°C. Neurons were mounted in an open chamber and were observed using a Leica DMI6000B microscope with a HC PL APO 20x NA0.75 objective. Fura-2-AM fluorescence was measured at 340 nm and 380 nm excitation, and 530 nm emission, DiI images were captured using a standard Leica RFP filter cube excitation 546nm and emission 605nm, using an Andor iXon 887 camera (Andor, Ireland) and MetaFluor v7.8.0 software (Molecular Devices). Neurons were challenged sequentially with AITC (100 µM), and either oleanolic acid (OA; 100 µM), Deoxycholic acid (DCA; 100 µM), 3-(2-chlorophenyl)-N-(4-chlorophenyl)-N,5-dimethyl-4-isoxazolecarboxamide (CCDC; 100 µM), taurolithocholic acid (TLCA; 100 µM), chloroquine (CQ; 10 µM) or BAM8-22 (2 µM), then capsaicin (1 µM), and KCl (50 mM). Images were analyzed using a custom journal in MetaMorph v7.8.2 software (Molecular Devices). For retrogradely labeled neurons, the DiI channel was separated, segmented, and binarized from this image using the Multi Wavelength Cell Scoring module on the basis of size and fluorescence intensity. Further analysis of the DiI positive cells of interest (< 25 µm diameter) were selected and responsive neurons were defined as those with an increase in fluorescence intensity standard deviation of >1.5 fold over the global baseline intensity standard deviation for all time points. DCA, CCDC, and TLCA, BAM8-22 were purchased from Cayman Chemicals (USA). CQ, AITC and Capsaicin were purchased from Sigma-Aldrich (Australia).

Visualisation of pERK activated neurons within the dorsal horn of the spinal cord following intra-colonic application of pruritogens and noxious colorectal distension (CRD).

Healthy control or CVH C57BL/6J mice or *Trpa1*$^{-/-}$ (1,2), *Tgr5*$^{-/-}$ (3,4) or *Tgr5-Tg* (3,4) mice were fasted overnight with free access to water and a 5% glucose solution, ensuring faecal pellets were absent from the colorectum for colorectal distension (CRD). In the first series of experiments mice were briefly anaesthetized with isoflurane anaesthetic and a 100 µl enema of CCDC (100 µM) or saline administered intra-colonically via a catheter. Subsequently, a 4 cm balloon catheter was inserted into the perianal canal and secured to the tail so that the start of the balloon sat 0.5 cm from the anal opening and the tube secured to the tail (7-9). The balloon catheter was attached to a sphygmomanometer pressure gauge and a 20ml syringe via a three-way stopcock. Mice were removed from the isoflurane chamber and upon regaining consciousness the balloon was distended for 10 seconds to a pressure of 40 mmHg, applied via the syringe. This pressure was released, and the balloon deflated (0 mmHg) for 5 seconds. This process was repeated 5 times, as per our previous studies (7-9,13). After the fifth distension, mice were injected with a euthanasia agent (0.2 ml / 250 g sodium pentobarbitone) and within 4 minutes underwent transcardial perfuse fixation with ice cold 4%

paraformaldehyde fixative. Our previous histological studies demonstrate that this protocol does not overtly damage the colonic epithelium. In a second series of experiments mice were briefly anaesthetized with isoflurane anaesthetic and a 100 μl enema of CCDC (100μM), CQ (10μM) or saline administered intra-colonically via a catheter. 10 minutes later, and without colorectal distension, mice were administered a euthanasia agent (0.125ml/250g sodium pentobarbitone) and underwent transcardial perfuse fixation. Mice were transcardially perfused with warm saline (0.85% physiological sterile saline) followed by ice-cold 4% paraformaldehyde in 0.1M phosphate buffer (Sigma-Aldrich, MO, USA).

In all studies following transcardial perfusion, thoracolumbar (T10-L1) spinal cord was removed, and post-fixed for 16 hours at 4°C in 4% PFA, then cryoprotected in 30% sucrose/0.1M phosphate buffer (Sigma-Aldrich) overnight at 4°C and then placed in 50% Optimal Cutting Temperature compound (OCT; Tissue-Tek, Sakura Finetek, CA, USA) in 30% sucrose/phosphate buffer solution for 7 hours, before block freezing in 100% OCT. Frozen sections (12µm) were cut using a cryostat and placed onto gelatin-coated slides. Immunohistochemistry for pERK was performed in a paired fashion, with tissue from healthy and CVH mice exposed to pruritogenic compound or saline run simultaneously. Sections were incubated with 5% normal chicken serum/0.2% Triton-X 200 (Sigma-Aldrich) in PBS (0.2% TX-PBS) for 30 minutes at room temperature to block non-specific binding of secondary antibodies. Sections were then incubated for 18 hours at room temperature with monoclonal anti-sera rabbit anti-phospho-p44/42 MAPK (Erk1/2),(Thr202/Tyr204) (D13.14.4E) (pERK; 1:200; #4370 Cell Signalling Technology, MA, USA; RIID: AB_2315112) diluted in 0.2 % TX-PBS . Sections were washed 3x with 0.2% TX-PBS before being incubated for 1 hour at room temperature with secondary antibody chicken anti-rabbit IgG (H+L) AlexaFluor-488 (1:200; #A-21441, Molecular Probes, ThermoFisher Scientific, MA, USA). Sections were then washed 3 times with 0.2% TX-PBS before mounting in ProLong Gold anti-fade (#P36930, ThermoFisher Scientific). Negative controls were prepared as above with the primary antibody omitted (13). The number of pERK-immunoreactive neurons for each mouse was obtained from >10 histological sections (50µm apart) from each spinal segment (T10-T12, T13-L1), visualised by fluorescence microscopy with 10x magnification. For quantification, dorsal horn neurons with intact nuclei and immunoreactivity above that of negative controls, where the primary antibody was omitted, were counted. Negative controls were prepared as above with the primary antibody omitted (13). Data represent Mean ± SEM and were analysed with unpaired t-tests using Prism 8 software (GraphPad Software, San Diego, CA, USA).

In vivo visceromotor responses (VMR) to colorectal distension (CRD) following intra-colonic administration of pruritogens

Noxious distension of the colorectum triggers the VMR, a nociceptive brainstem reflex consisting of the contraction of the abdominal muscles (30). Using abdominal electromyography (EMG),

this technique allows assessment of visceral sensitivity in vivo in fully awake animals (6,12,23,31). At least three days prior to the VMR and under isoflurane anaesthesia, the bare endings of two Teflon-coated stainless-steel wires (Advent Research Materials Ltd, Oxford, UK) were sutured into the right abdominal muscle of healthy control, *Tgr5*$^{-/-}$, *Mrgpr cluster*$^{-/-}$ or *Trpa1*$^{-/-}$ mice and tunnelled subcutaneously to be exteriorized at the base of the neck for future access. At the end of the surgery, mice received prophylactic antibiotic (Baytril®; 5mg/kg s.c.) and analgesic (buprenorphine; 0.4 mg/10 kg s.c.), were housed individually and allowed to recover. On the day of VMR assessment, mice were briefly anaesthetized using inhaled isoflurane and administered via a catheter a 100 μl enema consisting of the respective pruritogenic agonists. Overall, VMR was assessed in 11 cohorts of mice: **1)** Healthy mice with vehicle (saline), **2)** Healthy mice with intra-colonic CCDC (100μM), **3)** *Tgr5*$^{+/+}$ mice with intra-colonic CCDC (100μM), **4)** *Tgr5*$^{-/-}$ mice with intra-colonic CCDC (100μM), **5)** Healthy mice with intra-colonic CQ (10 μM), **6)** *Mrgpr cluster*$^{-/-}$ with intra-colonic CQ (10 μM), **7)** Healthy mice with intra-colonic BAM8-22 (20 μM), **8)** *Mrgpr cluster*$^{-/-}$ with intra-colonic BAM8-22 (20 μM), **9)** Healthy mice with an intra-colonic 'itch cocktail' consisting of a combination of CCDC (100μM), BAM8-22 (20μM) and CQ (10μM), **10)** *Trpa1*$^{-/-}$ mice with intra-colonic vehicle (saline) and **11)** *Trpa1*$^{-/-}$ mice with intra-colonic itch cocktail. Subsequently a lubricated balloon (2.5 cm length) was gently introduced through the anus and inserted into the colorectum up to 0.25 cm past the anal verge. The balloon catheter was secured to the base of the tail and connected to a barostat (Isobar 3, G&J Electronics, Willowdale, Canada) for graded and pressure-controlled balloon distension. Mice were allowed to recover from anaesthesia in a restrainer with dorsal access for 15 minutes prior to initiation of the distension sequence. Distensions were applied at 20-40-50-60-70-80 mmHg (20 second duration) at a 4 minute-interval so that the last distension was performed 30 min after intra-colonic administration of the itch cocktail of a combination of CCDC (100μM), BAM8-22 (20μM) and CQ (10μM). The EMG electrodes were relayed to a data acquisition system and the signal was recorded (NL100AK headstage), amplified (NL104), filtered (NL 125/126, Neurolog, Digitimer Ltd, bandpass 50–5000 Hz) and digitized (CED 1401, Cambridge Electronic Design, Cambridge, UK) to a PC for off-line analysis using Spike2 (Cambridge Electronic Design). The analogue EMG signal was rectified and integrated. To quantify the magnitude of the VMR at each distension pressure, the area under the curve (AUC) during the distension (20 seconds) was corrected for the baseline activity (AUC pre-distension, 20 s). Colonic compliance was assessed by applying graded volumes (40-200 μL, 20 s duration) to the balloon in the colorectum of fully awake mice, while recording the corresponding colorectal pressure as described previously (6,31,32). Following the final distension mice were humanely killed, by cervical dislocation. VMR data were presented as Mean ± SEM, where N represents the number of animals. VMR data were statistically analyzed by generalized estimating equations followed by LSD post hoc test when appropriate using SPSS 23.0. Data represent Mean ± SEM. Analysis and figures were prepared in Prism 8 software (GraphPad Software, San Diego, CA, USA).

In vivo assessment of animal behaviour

Behavioural testing was conducted on male healthy control mice (aged 12-14 weeks) with an average body weight of ~28 g (24 - 32g) on the experimental day. Mice were transferred in their home cage to a temperature-controlled test room (24 ± 1°C) and allowed to acclimatize for at least 10 min prior to testing. All experiments were performed during the light phase at the same time each day (between 13:30 and 17:00 hours). Locomotor activity and spontaneous behaviour in mice was evaluated using a behavioural spectrometer (Behavior Sequencer, Behavioral Instruments, NJ and BiObserve, DE) (33) which consisted of a 40 cm by 40 cm square arena enclosed at a height of 45 cm with a removable aluminum honeycomb sheet floor placed on vibration sensors and a miniature color CCD camera mounted in the ceiling of the enclosure above the center. In addition, the spectrometer was equipped with a row of 32 infrared transmitter and receiver pairs embedded in the walls at a height of 6.5 cm and halogen strip lights placed on the ceiling to illuminate the inside of the behavioural box. One side of the enclosure was a door allowing access to the area. For testing, healthy control or CVH mice were briefly anaesthetized with isoflurane anaesthetic and a 100 μl enema of an 'itch cocktail', consisting of CCDC (100 μM), BAM8-22 (20 μM) and CQ (10 μM), was administered intra-colonically via a lubricated catheter. A 100 μl saline enema was used as control. Mice were allowed to recover from anaesthesia (in a single cage with bedding) for 5 mins before the test was conducted. Subsequently, mice were individually placed in the centre of the behavioural spectrometer, and their behaviour was filmed/tracked and evaluated/analyzed by a computerized video tracking system (Viewer^3, BiObserve, DE) for 20 min. This software was used to evaluate/determine the total distance traveled in the open field, average velocity of locomotion, time spent in the central area (20 x 20 cm; considered to be an aversive place), wall distance (indication of anxiety; thigmotaxy = Behavior of walking next to the wall avoiding central areas), and different behavioral patterns like grooming, orienting, rearing, scratching and forms of locomotion. Grooming is a maintenance behaviour which represents the mouse licking its body and stroking its nose, head, ears with its forepaws. The same experimenter performed all experimental sessions and remained quiet and still during recordings in the same room. Testing was conducted in an order counterbalanced for treatment and time tested and mice were only used once and humanely culled after recordings (after behavioural testing) by CO_2 inhalation. Reported measurements occurred between 5-15 minutes after placement within the behavioural spectrometer. Between subjects, the apparatus was cleaned thoroughly with a disinfectant (F10 SC veterinary solution) to remove smell and excrement from previous mice. Normality of the data was assessed using the Shapiro-Wilk test prior to statistical analysis. Data represent Mean ± SEM and were analysed with unpaired two-tailed student's t-tests, using Prism 8 software (GraphPad Software, San Diego, CA, USA).

Human colonic biopsies

Fifteen healthy individuals, N=10 men (aged 24-55 years) and N=5 women (aged 18–47 years) were recruited primarily by community advertisement. Healthy controls had no personal or family history of

Irritable Bowel Syndrome (IBS), Inflammatory Bowel Disease (IBD) or other chronic pain conditions. Additional exclusion criteria for all subjects included infectious or inflammatory disorders, active psychiatric illness over the past 6 months assessed by structured clinical interview for the DSM-IV (MINI), use of corticosteroids in the past 6 months, use of narcotics, antidepressants, or other medications that could affect neuroendocrine function in the past 2 months, or current tobacco or alcohol abuse. Participants were compensated. Rectosigmoid biopsies were obtained by flexible sigmoidoscopy following tap water enemas. Specimens were flash-frozen in liquid nitrogen, and RNA was extracted with TRIzol reagent (ThermoFisher Scientific, Waltham, MA) (34).

Human dorsal root ganglia (DRG)

Thoracolumbar DRG (T9-L1) were acquired from 5 (3 female, 2 male) human adult donors (22.2 ± 2.08 years of age) during the removal of the vital organs for transplantation. The harvested DRG were immediately processed for down-stream RNA or Ca^{2+} imaging studies. Intact DRG from 4 donors were kept for QRT-PCR mRNA expression studies from each spinal level (T9,T10,T11,T12,L1) whilst additional DRG were dissociated as described previously to allow individual DRG neurons to be studied with single-cell-RT-PCR studies (7), or to allow Ca^{2+} imaging studies to be performed (7).

Quantitative RT-PCR analysis of pruritogenic ion channels and receptors in human colonic biopsies and human DRG

For human colonic biopsy studies, RNA was extracted from each biopsy using a PureLink RNA Mini kit (Invitrogen, Sydney, Australia, cat# 12183025) including an on-column DNAse treatment (LifeTechnologies, Sydney, Australia cat#12185-010) according to manufacturer's instructions. RNA was stored at -80°C in aliquots of 1-3 μL. Quantity and purity were checked using a NanoDrop spectrometer. RNA quality was assessed using a 2100 Bioanalyzer (Agilent) and all samples ranged from a RIN of 6 - 9. None of the measured samples showed a DNA peak in the profile.

For whole human DRG studies (7), RNA was extracted from individual T9-L1 DRG from 4 donors using a PureLink RNA Mini kit (Invitrogen, Sydney, Australia, cat# 12183025) including an on-column DNAse treatment (LifeTechnologies, Sydney, Australia cat#12185-010) according to manufacturer's instructions. RNA was stored at -80°C in aliquots of 1-3 μL. Quantity and purity were checked using a NanoDrop spectrometer. RNA quality was assessed using a 2100 Bioanalyzer (Agilent) and all samples ranged from a RIN of 6.5 - 9. None of the measured samples showed a DNA peak in the profile.

For both colonic biopsy and DRG RNA, qRT-PCR was performed using EXPRESS One-Step Superscript® qRT-PCR Kit reagents (Life Technologies) with commercially available hydrolysis probes

(TaqMan®; LifeTechnologies, Sydney, Australia, see **Supplementary Table 1**) and DEPC-treated water (AMBION cat# 11781-01K). Each reaction contained 10μl qPCR SuperMix, 1μL TaqMan® primer assay, 0.04μL ROX, 2μL water, 2μL SuperScript Reverse Transcriptase and 5μL RNA (20ng/well) in all experiments. Samples were run in duplicates. *GAPDH* was used as a reference gene and analysed on every plate together with the samples. The following cycle program was performed: cDNA synthesis 50°C for 15 minutess, Inactivation RT/activation polymerase 50°C for 90 sec, denaturation 95°C for 4 seconds extension 60°C for 30 seconds, repeated for 45 cycles. QRT-PCR curves were analysed using 7500Software v.2.06 from Life Technologies using their default baseline settings. The quantification cycle (Cq) value was set through the linear phase of the amplification curve. Cq-values were exported to Excel and relative abundance was estimated using delta Cq method. Values were then plotted in GraphPad Prism 8 (San Diego, CA, USA).

Single-cell PCR analysis of pruritogenic ion channels and receptors within individual human DRG neurons using RT-PCR

For single cell studies human DRG were dissociated, plated and single neurons picked using precision pulled and fire polished glass capillaries and a high-fidelity micromanipulator (7). A total of 53 human DRG neurons from 4 adult organ donors were individually picked and stored for subsequent single-cell-RT-PCR analysis. Only clearly single cells with no other cell or debris attached to it were picked. For RT-PCR experiments of single human DRG neurons DRG neurons, an Ambion® Single Cell-to-CT™ Kit (Life Technologies, Sydney, Australia) was used on an Applied Biosystems® 7500 Real-Time PCR System, with the TaqMan primers listed in **Supplementary Table 1**. The presence or absence of a target in an individual neuron was confirmed via cycle threshold analysis and GelRed™ Nucleic Acid Gel Stain (Biotium) gel-electrophoresis. Only cells expressing β-III-tubulin, a neuronal marker, without expressing *Gfap*, a glial marker, were included in the analysis.

$[Ca^{2+}]_i$ assays of human DRG neurons in response to pruritogens.

Experiments were conducted on isolated human DRG neurons from organ donors. Cells were loaded with 3 μM Fluo 8-AM (AAT Bioquest) containing 0.1 % Pluronic F-127 (Sigma) for 20 minutes. Extracellular solution contained in mM: 145 NaCl, 3 KCl, 2 $CaCl_2$, 1 $MgCl_2$, 10 HEPES, 10 glucose adjusted to pH 7.4 with NaOH. Fluo-8-loaded cells were excited at 480 nm and emissions at 520 nm were collected with a pcoEDGE sCMOS camera (PCO) mounted on an inverted microscope (Olympus IX71). Images were acquired at 0.2Hz for 3 minutes with the following protocol: 1 minute of baseline, 1 minute

of agonist application and 1 minute of washout. Agonists were applied in the following sequence and concentrations: 100 μM CCDC (BioVision), 1 μM Chloroquine (Sigma), 2 μM BAM8-22 (Tocris) and 2 μM NPFF (Tocris). An unrecorded 5 minutes washout period was applied between each agonist application. As positive controls, 100 nM Capsaicin (Sigma) and 50 μM AITC (Sigma) were applied for 20 seconds each at the end of the experiment, with a 20-minute washout interval between each application.

In order to mimic a pathological state, we incubated neurons with an "inflammatory soup" containing 10 μM each of Histamine (Sigma-Aldrich, Australia), PGE II (Tocris, UK), Serotonin (Tocris, UK) and Bradykinin (Sigma-Aldrich, Australia), 2 hours prior to the experiments at 37˚C. Experiments were conducted at room temperature. All agonists, except CCDC, were formulated as a stock solution using ddH_2O. CCDC was formulated in 100% DMSO (Sigma) and tested at a final DMSO concentration of 0.1 %. Image acquisition and data analysis were performed using MetaMorph software (Molecular Devices). Differences between the percentage of human neurons incubated in control media or media containing inflammatory mediators and responding to CCDC, CQ, BAM8-22, NPFF, Capsaicin and AITC states were analysed, and significance determine by Chi-square tests.

Statistics

Data are expressed as Mean ± SEM or the % of neurons/afferents. Figures were prepared in GraphPad Prism 8 Software (San Diego, CA, USA). N equals the number of animals, whilst n equals the number of neurons/afferents. Differences were considered significant at a level of **P*<0.05, ***P*<0.01, ****P*<0.001, *****P*<0.0001. VMR to CRD data were statistically analysed by generalised estimating equations followed by LSD post-hoc test using SPSS 23.0 (IMB, USA). All other data were analysed using GraphPad Prism 8 (San Diego, CA, USA) and analysed if the data were normally distributed using Kolmogorov-Smirnov or Shapiro-Wilk tests. These data were then analysed using either **1)** one-way ANOVA, with post hoc analysis conducted by making all possible comparisons among the treatment groups with the Tukey's tests, **2)** two-way ANOVA, with post hoc analysis conducted by making all possible comparisons among the treatment groups with Bonferroni tests, **3)** paired or **4)** unpaired 2-tailed t-tests or **5)** χ^2 analysis. The specific tests used to analyse each data set is indicated within the individual figure legends.

Study approval

The Animal Ethics Committees of the South Australian Health and Medical Research Institute (SAHMRI), Flinders University, The University of Adelaide and Monash University approved all experiments involving animals. All animal experiments conformed to the relevant regulatory standards and the ARRIVE guidelines.

All human tissues used for the study were obtained by legal consent from organ donors in the US. For DRG studies AnaBios Corporation's procurement network and includes only US based Organ Procurement Organizations and Hospitals. Policies for donor screening and consent are the ones established by the United Network for Organ Sharing (UNOS). Organizations supplying human tissues to AnaBios follow the standards and procedures established by the US Centres for Disease Control (CDC) and are inspected biannually by the Department of Health and Human Services (DHHS). Tissue distribution is governed by internal Institutional Review Boards (IRB) procedures and compliance with HIPAA regulations regarding patient privacy. All transfers of donor organs to ANABIOS are fully traceable and periodically reviewed by US Federal authorities. For human colonic biopsies study approval was obtained from the University of California, Los Angeles (UCLA, IRB# 12-001731) IRBs and all subjects signed a written informed consent form prior to starting the study.

Author contributions

J.C, L.G and S.M.B designed, performed and analysed the colonic afferent recordings. A.M.H, J.M, T.OD and S.M.B designed, performed and analysed the pERK dorsal horn studies. J.C, J.M, G.S and S.M.B designed, performed and analysed the VMR to CRD studies. G.S and S.M.B designed and performed the behavioural studies. TM.L, SG.C, N.W.B and S.M.B designed, performed and analysed the mouse single cell RT-PCR experiments. TM.L, D.P.P, N.W.B and S.M.B designed, performed and analysed the mouse Ca^{2+} imaging experiments. SG.C and S.M.B designed, performed and analysed the human DRG neuron single cell PCR and whole human DRG qRT-PCR expression studies. L.C collected and provided human colonic biopsies. X.D provided *Mrgpr cluster* $^{-/-}$ mice. A.L and S.M.B designed, performed and analysed the mouse colonic mucosal and the human biopsy qRT-PCR expression studies. P.M, A.G and S.M.B designed, performed and analysed the human DRG Ca^{2+} imaging studies. All authors contributed to the discussion and interpretation of the results. S.M.B wrote the manuscript with contributions and suggestions from all authors.

Acknowledgements

This work was supported by a National Health and Medical Research Council of Australia (NHMRC) Project Grants (#1083480 to S.M.B and D.P.P), an NHMRC R.D Wright Biomedical Research Fellow (APP1126378 to S.M.B), and an Australian Research Council (ARC) Discovery Early Career Research

Award (DE130100223 to A.M.H). NWB was supported by grants from the National Institutes of Health (NS102722, DE026806, DK118971) and the United States Department of Defence (W81XWH1810431).

SUPPLEMENTARY REFERENCES

1. Brierley SM, Castro J, Harrington AM, Hughes PA, Page AJ, Rychkov GY, et al. TRPA1 contributes to specific mechanically activated currents and sensory neuron mechanical hypersensitivity. *J Physiol.* 2011;589(Pt 14):3575-93.
2. Brierley SM, Hughes PA, Page AJ, Kwan KY, Martin CM, O'Donnell TA, et al. The ion channel TRPA1 is required for normal mechanosensation and is modulated by algesic stimuli. *Gastroenterology.* 2009;137(6):2084-95 e3.
3. Alemi F, Kwon E, Poole DP, Lieu T, Lyo V, Cattaruzza F, et al. The TGR5 receptor mediates bile acid-induced itch and analgesia. *J Clin Invest.* 2013;123(4):1513-30.
4. Alemi F, Poole DP, Chiu J, Schoonjans K, Cattaruzza F, Grider JR, et al. The receptor TGR5 mediates the prokinetic actions of intestinal bile acids and is required for normal defecation in mice. *Gastroenterology.* 2013;144(1):145-54.
5. Liu Q, Tang Z, Surdenikova L, Kim S, Patel KN, Kim A, et al. Sensory neuron-specific GPCR Mrgprs are itch receptors mediating chloroquine-induced pruritus. *Cell.* 2009;139(7):1353-65.
6. Castro J, Grundy L, Deiteren A, Harrington AM, O'Donnell T, Maddern J, et al. Cyclic analogues of alpha-conotoxin Vc1.1 inhibit colonic nociceptors and provide analgesia in a mouse model of chronic abdominal pain. *Br J Pharmacol.* 2018;175(12):2384-98.
7. Castro J, Harrington AM, Garcia-Caraballo S, Maddern J, Grundy L, Zhang J, et al. alpha-Conotoxin Vc1.1 inhibits human dorsal root ganglion neuroexcitability and mouse colonic nociception via GABAB receptors. *Gut.* 2017;66(6):1083-94.
8. Castro J, Harrington AM, Hughes PA, Martin CM, Ge P, Shea CM, et al. Linaclotide inhibits colonic nociceptors and relieves abdominal pain via guanylate cyclase-C and extracellular cyclic guanosine 3',5'-monophosphate. *Gastroenterology.* 2013;145(6):1334-46 e1-11.
9. de Araujo AD, Mobli M, Castro J, Harrington AM, Vetter I, Dekan Z, et al. Selenoether oxytocin analogues have analgesic properties in a mouse model of chronic abdominal pain. *Nat Commun.* 2014;5:3165.
10. Osteen JD, Herzig V, Gilchrist J, Emrick JJ, Zhang C, Wang X, et al. Selective spider toxins reveal a role for the Nav1.1 channel in mechanical pain. *Nature.* 2016;534(7608):494-9.
11. Hughes PA, Brierley SM, and Blackshaw LA. Post-inflammatory modification of colonic afferent mechanosensitivity. *Clin Exp Pharmacol Physiol.* 2009;36(10):1034-40.
12. Grundy L, Harrington AM, Castro J, Garcia-Caraballo S, Deiteren A, Maddern J, et al. Chronic linaclotide treatment reduces colitis-induced neuroplasticity and reverses persistent bladder dysfunction. *JCI Insight.* 2018;3(19).
13. Harrington AM, Brierley SM, Isaacs N, Hughes PA, Castro J, and Blackshaw LA. Sprouting of colonic afferent central terminals and increased spinal mitogen-activated protein kinase expression in a mouse model of chronic visceral hypersensitivity. *J Comp Neurol.* 2012;520(10):2241-55.
14. Adam B, Liebregts T, Gschossmann JM, Krippner C, Scholl F, Ruwe M, et al. Severity of mucosal inflammation as a predictor for alterations of visceral sensory function in a rat model. *Pain.* 2006;123(1-2):179-86.
15. Hughes PA, Castro J, Harrington AM, Isaacs N, Moretta M, Hicks GA, et al. Increased kappa-opioid receptor expression and function during chronic visceral hypersensitivity. *Gut.* 2014;63(7):1199-200.

16. Cattaruzza F, Lyo V, Jones E, Pham D, Hawkins J, Kirkwood K, et al. Cathepsin S is activated during colitis and causes visceral hyperalgesia by a PAR2-dependent mechanism in mice. *Gastroenterology.* 2011;141(5):1864-74 e1-3.
17. Brierley SM, Jones RC, 3rd, Gebhart GF, and Blackshaw LA. Splanchnic and pelvic mechanosensory afferents signal different qualities of colonic stimuli in mice. *Gastroenterology.* 2004;127(1):166-78.
18. Brierley SM, Carter R, Jones W, 3rd, Xu L, Robinson DR, Hicks GA, et al. Differential chemosensory function and receptor expression of splanchnic and pelvic colonic afferents in mice. *J Physiol.* 2005;567(Pt 1):267-81.
19. Bellono NW, Bayrer JR, Leitch DB, Castro J, Zhang C, O'Donnell TA, et al. Enterochromaffin Cells Are Gut Chemosensors that Couple to Sensory Neural Pathways. *Cell.* 2017;170(1):185-98 e16.
20. Brookes SJ, Spencer NJ, Costa M, and Zagorodnyuk VP. Extrinsic primary afferent signalling in the gut. *Nat Rev Gastroenterol Hepatol.* 2013;10(5):286-96.
21. Hughes PA, Harrington AM, Castro J, Liebregts T, Adam B, Grasby DJ, et al. Sensory neuro-immune interactions differ between irritable bowel syndrome subtypes. *Gut.* 2013;62(10):1456-65.
22. Brierley SM, Page AJ, Hughes PA, Adam B, Liebregts T, Cooper NJ, et al. Selective role for TRPV4 ion channels in visceral sensory pathways. *Gastroenterology.* 2008;134(7):2059-69.
23. Jimenez-Vargas NN, Pattison LA, Zhao P, Lieu T, Latorre R, Jensen DD, et al. Protease-activated receptor-2 in endosomes signals persistent pain of irritable bowel syndrome. *Proc Natl Acad Sci U S A.* 2018;115(31):E7438-E47.
24. Brierley SM, Jones RC, 3rd, Xu L, Gebhart GF, and Blackshaw LA. Activation of splanchnic and pelvic colonic afferents by bradykinin in mice. *Neurogastroenterol Motil.* 2005;17(6):854-62.
25. Salvatierra J, Castro J, Erickson A, Li Q, Braz J, Gilchrist J, et al. NaV1.1 inhibition can reduce visceral hypersensitivity. *JCI Insight.* 2018;3(11).
26. Beyak MJ. Visceral afferents - determinants and modulation of excitability. *Auton Neurosci.* 2010;153(1-2):69-78.
27. Grundy L, Chess-Williams R, Brierley SM, Mills K, Moore KH, Mansfield K, et al. NKA enhances bladder-afferent mechanosensitivity via urothelial and detrusor activation. *Am J Physiol Renal Physiol.* 2018;315(4):F1174-F85.
28. Hughes PA, Brierley SM, Young RL, and Blackshaw LA. Localization and comparative analysis of acid-sensing ion channel (ASIC1, 2, and 3) mRNA expression in mouse colonic sensory neurons within thoracolumbar dorsal root ganglia. *J Comp Neurol.* 2007;500(5):863-75.
29. Lieu T, Jayaweera G, Zhao P, Poole DP, Jensen D, Grace M, et al. The bile acid receptor TGR5 activates the TRPA1 channel to induce itch in mice. *Gastroenterology.* 2014;147(6):1417-28.
30. Ness TJ, and Gebhart GF. Colorectal distension as a noxious visceral stimulus: physiologic and pharmacologic characterization of pseudaffective reflexes in the rat. *Brain Res.* 1988;450(1-2):153-69.
31. Carstens BB, Berecki G, Daniel JT, Lee HS, Jackson KA, Tae HS, et al. Structure-Activity Studies of Cysteine-Rich alpha-Conotoxins that Inhibit High-Voltage-Activated Calcium Channels via GABA(B) Receptor Activation Reveal a Minimal Functional Motif. *Angew Chem Int Ed Engl.* 2016;55(15):4692-6.
32. Deiteren A, De Man JG, Ruyssers NE, Moreels TG, Pelckmans PA, and De Winter BY. Histamine H4 and H1 receptors contribute to postinflammatory visceral hypersensitivity. *Gut.* 2014;63(12):1873-82.
33. Inserra MC, Israel MR, Caldwell A, Castro J, Deuis JR, Harrington AM, et al. Multiple sodium channel isoforms mediate the pathological effects of Pacific ciguatoxin-1. *Sci Rep.* 2017;7:42810.
34. Videlock EJ, Mahurkar-Joshi S, Hoffman JM, Iliopoulos D, Pothoulakis C, Mayer EA, et al. Sigmoid colon mucosal gene expression supports alterations of neuronal signaling in irritable

bowel syndrome with constipation. *Am J Physiol Gastrointest Liver Physiol.* 2018;315(1):G140-G57.
